# Factors Affecting Site Use Preference of Grazing Cattle Studied from 2000 to 2020 through GPS Tracking: A Review

**DOI:** 10.3390/s21082696

**Published:** 2021-04-11

**Authors:** M. Jordana Rivero, Patricia Grau-Campanario, Siobhan Mullan, Suzanne D. E. Held, Jessica E. Stokes, Michael R. F. Lee, Laura M. Cardenas

**Affiliations:** 1 Sustainable Agriculture Sciences, Rothamsted Research North Wyke, Okehampton EX20 2SB, Devon, UK; patricia.grau@rothamsted.ac.uk (P.G.-C.); MRFLee@harper-adams.ac.uk (M.R.F.L.); laura.cardenas@rothamsted.ac.uk (L.M.C.); 2 Bristol Veterinary School, University of Bristol, Langford BS40 5DU, Somerset, UK; siobhan.mullan@ucd.ie (S.M.); suzanne.held@bristol.ac.uk (S.D.E.H.); jessica.stokes@rau.ac.uk (J.E.S.); 3 School of Veterinary Medicine, University College Dublin, Belfield, Dublin 4, Ireland; 4 School of Agriculture, Food and the Environment, Royal Agricultural University, Stroud, Cirencester GL7 6JS, Gloucestershire, UK; 5 Harper Adams University, Edgmond, Newport TF10 8NB, Shropshire, UK

**Keywords:** cattle distribution, grazing patterns, landscape use, sustainable grazing, site selection, grazing livestock

## Abstract

Understanding the behaviour of grazing animals at pasture is crucial in order to develop management strategies that will increase the potential productivity of grazing systems and simultaneously decrease the negative impact on the environment. The objective of this review was to summarize and analyse the scientific literature that has addressed the site use preference of grazing cattle using global positioning systems (GPS) collars in the past 21 years (2000–2020) to aid the development of more sustainable grazing livestock systems. The 84 studies identified were undertaken in several regions of the world, in diverse production systems, under different climate conditions and with varied methodologies and animal types. This work presents the information in categories according to the main findings reviewed, covering management, external and animal factors driving animal movement patterns. The results showed that some variables, such as stocking rate, water and shade location, weather conditions and pasture (terrain and vegetation) characteristics, have a significant impact on the behaviour of grazing cattle. Other types of bio-loggers can be deployed in grazing ruminants to gain insights into their metabolism and its relationship with the landscape they utilise. Changing management practices based on these findings could improve the use of grasslands towards more sustainable and productive livestock systems.

## 1. Introduction

Understanding the behaviour of grazing animals at pasture, i.e., how grazing animals distribute themselves and move across pasture and what activities they perform in each area, is crucial in order to develop management strategies that will increase the potential productivity of the grazing systems and also decrease their negative impact on the environment (nutrient losses to water and gaseous emissions). Therefore, it is necessary to record and study their movements in space and time to reveal the motivation for their site use preferences. This would enable the design and management of more sustainable grazing systems. For instance, high livestock densities, resulting in greater urine and faecal deposition, lead to a greater concentration and an uneven distribution of soil nutrients (mainly of nitrogen (N), phosphorus (P), and potassium (K)) [1,2]. There is a potential for a nutrient build up (hotspot) caused by the excreta of livestock at pasture by preferential use of certain areas of the field. If these hotspot areas are identified and the characteristics of that particular area that are influencing animal preference for its use are determined, targeted mitigation managements [3,4] to reduce gaseous emissions and water pollution [5,6,7,8] resulting from the hotspot can be designed. For example, welfare-friendly (as defined within the Five Freedoms [9]) interventions can be put in place to encourage a more even distribution of cattle in the field and decrease their impact on soil conditions and emissions, e.g., strategically positioning water troughs, supplemental feed stations and shade in the paddocks. Apart from environmental stewardship (reducing gaseous emissions and pollutants to water courses) and production efficiency (improving access of high quality feed) [10], real-time GPS tracking and animal biologging can be used to detect disease and/or welfare concerns remotely (e.g., a longer-than-normal time spent by livestock near the water troughs may indicate a failure in the water system) [11]. The information aligned to these three benefits (environment, production and welfare) could be integrated into a precision livestock approach [12] representing an integrative tool towards improving the sustainability of ruminant grazing systems through designing “smart farms”.

Animal tracking and monitoring technology has progressed markedly over the past five decades [13]. The study of animal behaviour has evolved from the initial naturalists’ direct visual observations to the use of electronic devices [13]. The main technologies for monitoring livestock in the field currently are GPS, radio tracking, and wireless local area network, although there are some other tools (e.g., Bluetooth, ultrasound) that can be deployed for indoor monitoring [14]. Additionally, global navigation satellite systems (GNSS) have the potential to be used for animal positioning [15].

GPS tracking data can be enhanced when the location information is complemented with the corresponding activity assigned to it, as this allows the mapping of the different activities (e.g., grazing, resting) to different areas within the pasture [16]. Therefore, more recently approaches have advanced further to make use of biologging such as accelerometers and magnetometers [17]. This provides the user with new tools to understand the reasons behind the individual and “group” decision-making through the identification and quantification of animal behaviour at a temporal and spatial scale. Crucially, Manning et al. [18] found that wearing the collars required for attaching the GPS devices does not modify the normal animal behaviour when tracking devices are used to investigate cattle location, and that a habituation period seemed not to be required when using similar equipment in cattle studies. This knowledge validated the use of GNSS/GPS technology for studying cattle grazing behaviour.

Swain et al. [16] reviewed the use of GPS tracking for wildlife and domesticated livestock and summarised the history of its use, highlighted ideas for the future use of GPS tracking data, explored opportunities to use spatial statistics to identify group behaviours, and linked behavioural preferences with landscape evaluation. The authors performed a wide Web of Science search for GPS studies over the 20-year period between 1990–2009 and retrieved 139 articles. However, fewer than 20 articles were cited in their work discussing the use of GPS tracking for behavioural classification and animal preference. Even thought it was not the focus of their review, Swain et al. [16] stated that “understanding how behavioural preferences, such as grazing, relate to spatially constrained environmental factors, such as herbage quality, is an emerging opportunity for GPS-based behavioural classification”. We performed a similar search (on 20 April 2020) using the terms “GPS AND (wildlife OR livestock)” and identified 827 journal papers published between 2010 and 2019 (201 livestock only, 567 wildlife only, and 59 both livestock and wildlife) with a sustained increase in the total number of articles published per year since the initial review of Swain et al. [16]. While the current search does not give a definitive number of all studies dealing with the use of GPS for monitoring animal movements, it gives a clear indication of the remarkable increase in these types of studies in the last decade.

Through these new techniques, it has been possible to identify and analyse with a higher level of detail the factors influencing behaviour (e.g., water sources position, stocking rate, type of pasture, topography, breed) in a diverse range of landscapes and environments. Thus, this technology has contributed to a greater understanding and characterisation of the impact of cattle distribution on the environment. Agreeing on the knowledge gap recognised by Swain et al. [16], and considering the substantial increase in the number of papers published reporting the use of GPS tracking in livestock in the last decade, we identified the need to assemble the existing literature with a focus on factors driving the distribution patterns in the field of grazing cattle to inform targeted management strategies. The scope of this review does not cover the technical details of the GPS devices, nor those of the other bio-loggers available for animal monitoring, but we have focused on the application of GPS sensors to understand cattle site use preference and the knowledge generated from the numerous studies published since 2000. This review therefore summarizes and analyses the scientific literature (research articles) that have addressed the site use preference of grazing cattle using GPS devices in the past 21 years (2000–2020).

## 2. Selection of Studies and Their Main Characteristics

**Figure 1:**
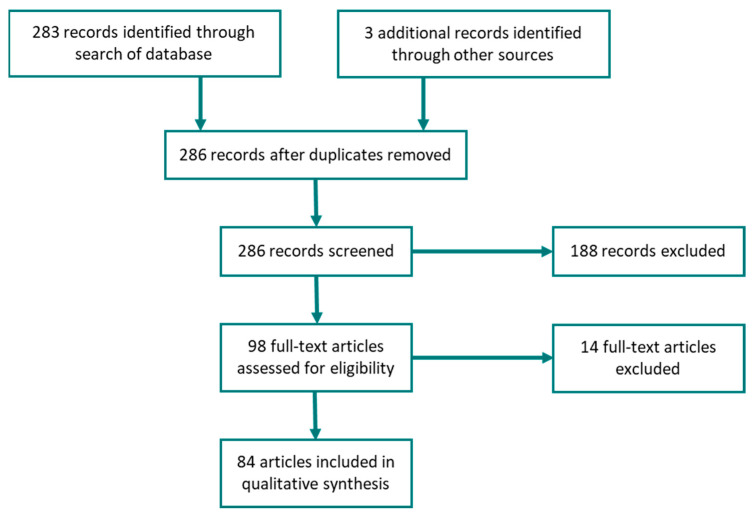
Systematic screening process for eligibility of articles included in this review.

A Web of Science search (on 11 May 2020) using the criteria “topic: ((GPS or GNSS) and (cattle or cow *) and (distribution or field use or site use or landscape use or grazing patterns or site selection or cattle location or landscape selectivity or occupation pattern or patterns of livestock activity)) and document type: (Article OR Note)” identified 251 journal articles published between 2000 and 2019. An additional search (on 15 March 2021) identified 32 articles published in 2020; therefore, a total of 283 articles published between 2000 and 2020 were identified. By screening the abstract, 188 articles were excluded due to the following criteria: the animals’ species was not cattle (79); the variables used did not include the spatial distribution of cattle (68); the studies did not report the use of GPS (8) or the GPS devices were not attached to animals (31); or the articles were not written in English (2). This exclusion resulted in an initial selection of 95 articles. After reading the papers, 14 additional articles were excluded because the studies did not assess the spatial distribution of cattle or because the GPS devices were not attached to the animals (they were mainly focused on device testing, modelling or methodological development). Three additional relevant articles were identified from the references used in the selected articles. In total, 84 research articles (87 studies), 22 from 2000 to 2009, 53 from 2010 to 2019, and 9 published in 2020 were included in the analysis. This systematic process is represented in Figure 1 (Supplementary Table S1 shows a list of the articles used).

To condense the relevant information of the selected studies, a summary table was produced (Supplementary Table S1) with the following headings: reference, starting year, ending year, type of system, climate, country, average size of the fields used, average herd size, average number of cattle tracked, average percentage of cattle tracked, animal category, frequency of GPS recording, categories of factors assessed and categories of response variables measured.

**Figure 2:**
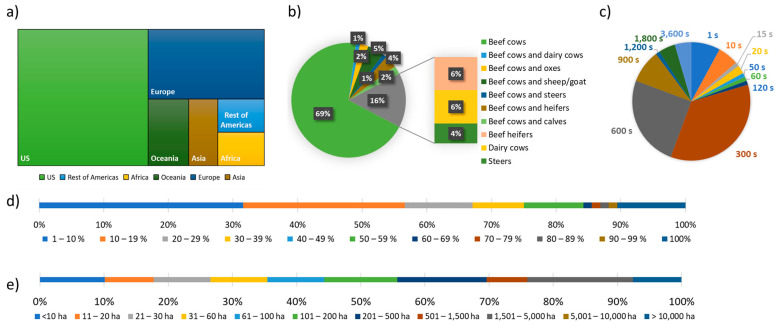
Descriptive statistics of the main characteristics of the 84 studies published between 2000 and 2020 on the use of GPS tracking on cattle to determine the factors affecting their site use preference on grazing systems: (**a**) proportion of studies per region; (**b**) percentage of animal types; (**c**) frequency with which GPS fixes were taken from the collared animals; (**d**) percentage of animals tracked within the herd; (**e**) percentage of paddock size categories.

On average, 2.2 studies per year were published between 2000 to 2009, increasing to 5.3 between 2010 to 2019, whilst in 2020 nine studies were published in that one year, showing the clear trend of increasing the number of studies published across time. Figure 2 shows the breakdown of the studies included in this review in relation to their region (Figure 2a), animal type/s (Figure 2b), frequency of GPS fixes (Figure 2c), percentage of animals tracked (Figure 2d), and paddock size (Figure 2e). Most of the studies (53%) were carried out in the US, followed by Europe (24%) and Oceania (8%), whilst the rest of the Americas, Asia and Africa each accounted for approximately 5–6% of the studies (Figure 2a), with 69% of the studies taking place over 1 or 2 years. The majority of the research reported was performed on beef cattle (94%), mainly in cows (84%) (Figure 2b), and the animals were most frequently tracked with GPS signals sent every 5 to 15 min (69%) (Figure 2c). For 57% of the studies GPS collars were put on less than a fifth of the herd, whereas 11% tracked the whole herd (Figure 2d). Of the reports informing the size of the experimental areas, only 18% used fields with up to 20 ha in size, whereas 30% of the studies were carried out in fields with a size greater than 500 ha (Figure 2e).

**Figure 3:**
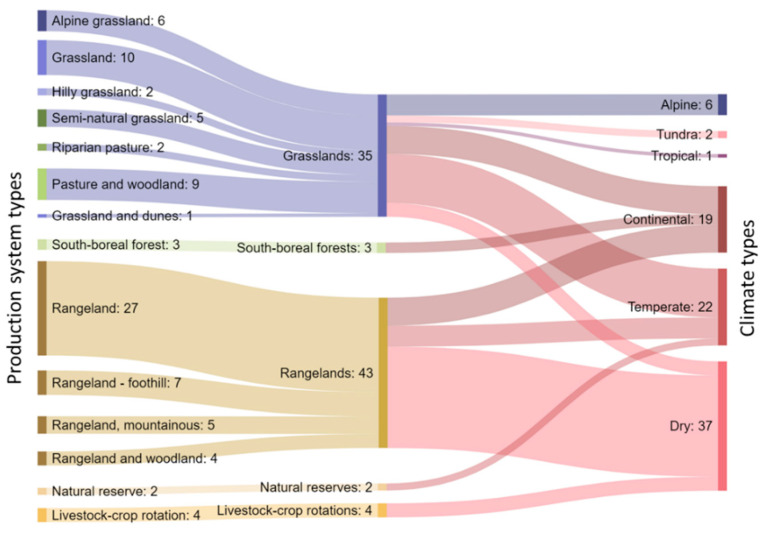
Number of production systems and climate types on the study sites from the 84 studies (87 experiments in total) published between 2000 and 2020 on the use of GPS tracking on cattle to determine the factors affecting their site use preference on grazing systems.

Figure 3 shows the relationship between the production systems and climate types. Regarding the conditions in which the studies were undertaken, 43% were conducted in dry climates (desert, arid, semiarid), 25% in temperate conditions and 22% in continental climatic conditions (Figure 3). Almost half of the studies were performed in rangeland production systems (average size of the research area: 1600 ha), whilst grassland-based systems accounted for 29% of the studies (average area: 51 ha), although some studies combined grasslands and woodlands or dunes (11%) (Figure 3).

**Figure 4:**
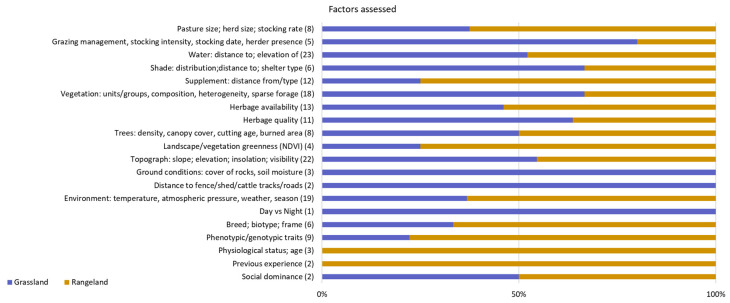
Categories of factors assessed in studies published between 2000 and 2020 on the use of GPS tracking on cattle to determine the factors affecting their site use preference on grazing systems (numbers between brackets refer to the total number of studies carried out only in grasslands and rangelands that included the assessment of each factor class).

**Figure 5:**
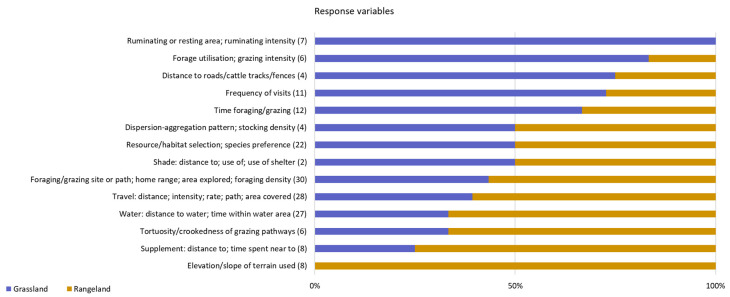
Response variables assessed in studies published between 2000 and 2020 on the use of GPS tracking on cattle to determine the factors affecting their site use preference on grazing systems (numbers between brackets refer to the total number of studies carried out nly in grasslands and rangelands that included each response variable class).

These figures highlight the diversity of experimental conditions and systems analysed in this review, with a preponderance of rangeland systems, particularly under dry environments, and with a remarkable difference in size of the research areas between rangeland and grasslands. This preponderance towards rangeland systems in dry environmental conditions may have imposed a bias in the factors assessed related with size of the grazing area or number and distribution of “rewards” such as supplemental feed (Figure 4). Other factors that were assessed in greater proportion on the rangeland systems are herbage availability (either measured directly or by greenness index) and environmental conditions. On the other hand, for studies carried out in grasslands, the most relevant factors were vegetation types and herbage quality (Figure 4). When analysing the response variables, the site use preference was primarily assessed by foraging or grazing areas, travel characteristics, proximity to water points and resources or habitats selection, with the first three groups of variables being more frequently studied in the rangeland conditions (Figure 5). Intuitively, these variables seem to be more relevant in larger areas.

Overall, extensive systems of beef cows producing in dry conditions, mainly in the US, have been the predominant focus of these type of studies. It is logical to think that an even distribution and resource use of cattle in large grazing areas may be critical under extensive production circumstances. However, with the increasing need to improve the sustainability of grazing livestock systems as a means of ensuring global food security with reduced environmental impact, it also seems crucial to focus on the efforts in other relevant systems (e.g., grasslands, silvopastoral) and environmental conditions (e.g., temperate, tropical), and even in other animal types (e.g., dairy cows, growing beef steers and heifers) that also play a significant role globally in pasture-based food production. This reoriented focus would benefit from combining GPS devices with other bio-loggers to gain insights into grazing behaviour and its relationship with the systems’ performance and animal health. In the following sections, we analysed the studies published between the years 2000 and 2020 in relation to the relevant factors affecting the distribution of cattle in grazing lands.

## 3. Effect of Stocking Rate and Grazing Method on Site Use Preference

Stocking rate (SR), which is the animal-to-land ratio measured over a defined period, is a more consistent and persuasive management variable than grazing method to influence animals’ spatial use of the fields [19]. Along with pasture size, these management strategies can influence the proportion of time grazing cattle make use of certain areas (e.g., time spent near water points) [8]. Overall, the spatial variation in cattle distribution in a paddock, either moderated by SR or other factors, is a key variable affecting the pattern of herbage utilisation in extensive systems [20]. For instance, cattle under high SR spend more time grazing and make more use of less favourable areas in woodlands such as woody vegetation and steeper slopes compared to moderate SR in the Australian savanna [21]. Stocking rate also affects grazing distances; from fences, water troughs, and supplementary feed stations, since animals graze further from these points [21] and walk longer distances daily, i.e., exploring larger daily areas [22] as the SR increases.

With regard to grazing methods, the implementation of rotational grazing (RG) with greater SR in large paddocks improves the distribution of cattle grazing through an increased utilisation of zones that were previously sporadically grazed under continuous stocking (CS) and at lower SR [23]. The less clustered nature of the grazing pattern in RG systems suggests that this grazing method could be a good management tool to improve the use of grasslands [23]. Intuitively, we can assume that the larger the grazing area the more important the need to apply some management strategy to improve the distribution of cattle in the area. This could be the case in the large mountainous pastures (~2400 ha), where Rinella et al. [24] tested the strategy of shifting the stocking location each year (between south and east) and found that certain regions were grazed much more heavily than others (clustered grazing) though the strategy greatly decreased the grazing of the overused regions.

**Figure 6:**
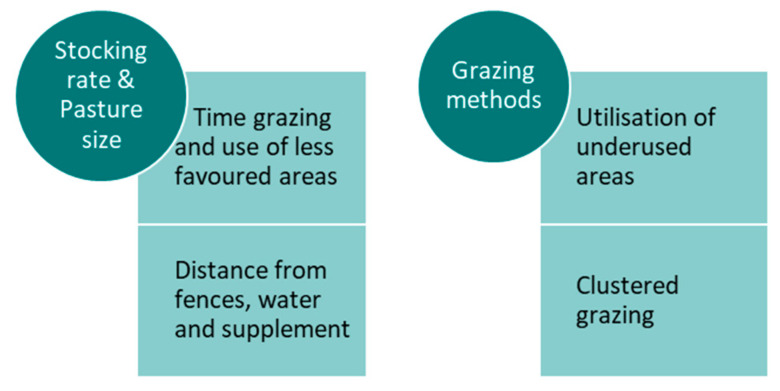
Summary diagram of the main variables of site use preference being affected by the management factor.

Hence, SR, grazing method, and paddock size should be considered along with field shape and features when evaluating the temporal/spatial distribution of grazing cattle [8] in order to implement interventions to improve the site use. However, it seems that more studies are needed to test the effect of SR and grazing methods on a wider range of paddock sizes and environments to be able to tailor these strategies to the different production circumstances. A summary diagram of the main variables being affected by the management factor is shown in Figure 6.

## 4. Effect of External Factors on Site Use Preference

In this section, the main factors analysed were: (i) water location (and number of water points), which interacts with environmental conditions; (ii) access to shade and shelter in relation to environmental conditions, which in turn are also related with water points visits; (iii) supplemental feed location and type, which interacts with grazing management; (iv) vegetation characteristics, the effect of which is mainly driven by animal selectivity and covers aspects such as forage quality and availability, botanical composition and the heterogeneity of the distribution of the different vegetation types, and their interactions with other features of the field; and (v) landscape characteristics such as topography (e.g., slope) and ground conditions. Even though all these factors interact among each other, we are presenting each main factor in the following subsections to ease the understanding of their individual effects.

### 4.1. Water Location

Cattle’s drinking can be variable across the day, i.e., 1–11 times per day, varying with biotype and climate conditions, and restrictions in access to water can modify animal behaviour and reduce performance (e.g., live weight gain) [25]. Water is considered the single most significant factor of livestock distribution at a paddock scale and can influence landscape use preference [20,26,27]. Actually, the elevation of the watering sites and the horizontal distance to the water are significant predictors of livestock grazing distribution (these two variables negatively correlate to the frequency of visits to the water sources) [28]. Grazing animals typically prefer to ruminate and rest in small areas close to water troughs [10] or trees [29], although grazing hotspots are also recognised near to water points [10,29].

Given the dependence of cattle’s water needs on environmental conditions, the effect of the water points’ locations and number on cattle distribution can vary across diverse production circumstances. In Italian alpine grasslands (uplands), proximity to water affects grazing distribution only under the RG [23]. In a subtropical Australian savanna, the spatial distribution of the cattle is not homogeneous and seems to be greatly affected by the prevalent drought conditions and location of water sources within the paddocks (~105 ha) [20]. Thus, setting up additional pasture subdivisions and the provision of additional water sources in large paddocks can improve the homogeneity of the grazing distribution by enabling the livestock to scatter more widely over a paddock [30]. In smaller paddocks in rangelands (20–32 ha paddocks), the percentage of time spent near water sources (troughs and ditches) in warm seasons is double the time than in cold seasons, with some exceptions under higher temperatures in winter [31]. On the other hand, the supply of additional off-stream water sources shows inconsistent results in its effect on cattle’s use of riparian areas [5,6,7,32], whereas fencing streams decreases the time cattle spend grazing in riparian areas [5].

Therefore, in general, the provision of additional water points in grasslands will improve the distribution of cattle across the paddock. However, in extensive cattle grazing areas (>1000 ha) this might not be as effective as in grasslands (~100 ha or less). For instance, in a rangeland system (~14,600 ha), when watering sources are dispersed over the landscape some watering points are highly utilised while others are infrequently used [33], especially during drier seasons. The water sources that are used most frequently are reachable from highly utilised cattle focal points on the landscape. This would imply that there are other factors defining the preferred areas of the landscape and the selection of water points used by cattle is determined by its proximity. Additionally, this preference for locations near to water sources can change throughout the season; in heterogeneous mountain rangelands, cattle stay near permanent streams and water sources in the dry–cold season, whereas this preference is not observed in the wet–warm season [34]. It is clear that the selection of water sources used, and the frequency of visits is affected by the ambient conditions. This link is addressed in the following section where the access to shade and shelter is discussed in relation to weather conditions.

**Figure 7:**
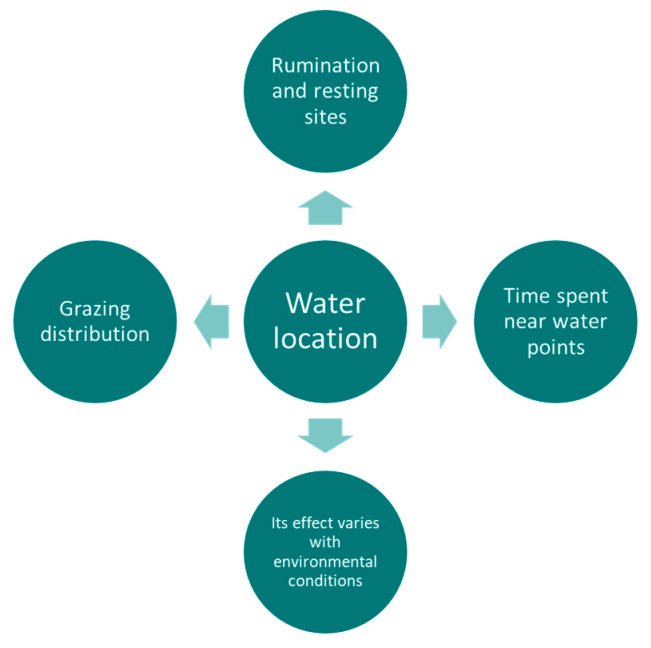
Summary diagram of the main variables of site use preference being affected by the location of water points.

This highlights the complex nature of extensive rangeland when it comes to defining the optimal design of the landscape features that influence livestock site use preference, particularly additional water points. On the other hand, little work has been carried out in more intensive beef cattle production systems where the sizes of the fields can be smaller (<10 ha). These two extremes production system types (extensive rangeland and intensive grassland) would require further work to better understand the interactions between water points’ location, type and number with landscape characteristics and its effect on cattle site use preference. Figure 7 shows a summary of the main variables being affected by the location of the water points.

### 4.2. Access to Shade and Shelter in Relation to Climate and Weather

One of the most important external factors impacting animal behaviour is the weather [35], and the ambient variable that has been most commonly associated with cattle site use preference is air temperature. In temperate climate areas, the air temperature in summer can rise above the upper critical temperature limit causing heat-stress in livestock, and higher solar radiation and relative humidity can increase the heat load [36]. Cattle need shade to ease thermoregulation under these conditions, which can determine their location within the field. Therefore, shade is an important factor in determining the spatial patterns of grazing animals, particularly in summer [37,38], and this is more pronounced when the shade is located near to water sources [8].

**Figure 8:**
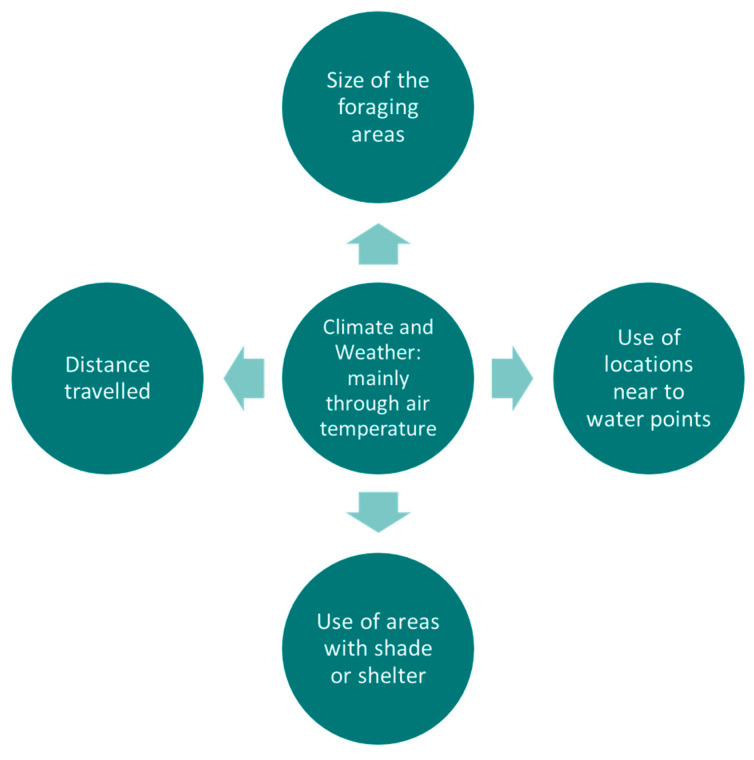
Summary diagram of the main variables of site use preference being affected by climate and weather conditions.

Heat stress factors influence the distance of cattle to the river and riparian zones in grassland from continental [6,7] and temperate climates [32] since riparian shade or water are more attractive during periods of elevated temperatures, solar radiation and windspeed, and lower relative humidity [32]. Overall, ambient temperature is superior to the other microclimatic variables in predicting cattle presence in shade [7]. However, in subtropical grasslands the effect of ambient temperature has not been detected [38]. Similarly, cattle grazing in south-boreal forests in Norway (continental climate) are not affected by sun exposure when selecting grazing and resting sites [39], maybe due to the occurrence of mild temperatures during the study period. This lack of uniform temperature effects on selection of feeding location may be a result of varying levels of heat stress during hot seasons, which may provoke cattle to display more subtle heat stress-reducing behaviours, e.g., altered orientation to minimise exposure to solar radiation or maximize exposure to winds [38]. Figure 8 shows a summary of the main variables being affected by environmental conditions, particularly temperature.

Increasing short-term thermal stress is also associated with a detectable increase in woodland preference by British breed cows in semiarid rangelands [40]. Additionally, a seasonal variation in the use of shade has been observed in semiarid rangelands; lowest in summer and spring and highest in autumn and winter [41], which contrasts with previous studies in other climates. Even in mild winters, cattle in nature reserves in temperate climates increasingly avoid open areas and seek shelter from an apparent temperature of around 0 °C (especially so during night-time) [42], or at high heat-load in summer [43]. Cattle also tend to seek shelter in the trees in semiarid rangelands during cold and rainy days [22] and have been shown to walk longer distances searching for shelter during snowstorms [44]. Decreasing temperatures are also associated with longer postsunset distances travelled, and less sinuous search patterns [22]. Straighter movement paths are also associated with rainy weather and winds from the W–NW for predawn night-time hours, and stronger winds for daytime hours [22]. Moreover, as precipitation increases, cows spend less time grazing in each patch and return to grazed patches more often [45], whereas daily distance travelled is greater and foraging area is expanded during periods with higher precipitation in desert rangelands [46]. Additionally, a positive correlation between daily walking distance of cattle and atmospheric pressure was found in rangelands (continental climate) [35].

Interestingly, cattle have a differential preference for natural and artificial shelter [43]. When natural shelter is sparse, a more dispersed distribution of it tempers the increased use of shelter by cattle with increasing heat-load in summertime, and, when adequately available, cattle prefer natural to artificial shelter. However, when insufficient natural shelter is available, cattle use the artificial shelter, especially with increasing heat load [43] or cold conditions [42]. During high heat load, numerous meteorological variables and indices differ between open area, natural and artificial shelter, in providing respite from conditions more effectively, which may explain the differential use of these areas. Additionally, vigilance against predators may be a factor influencing cattle’s preferential use of more open natural shelter versus an artificial shelter with three closed flanks [43], even at the expense of forage intake. In fact, animals may give up travelling longer distances to obtain new feed resources when those locations may have a higher probability of danger [47].

**Figure 9:**
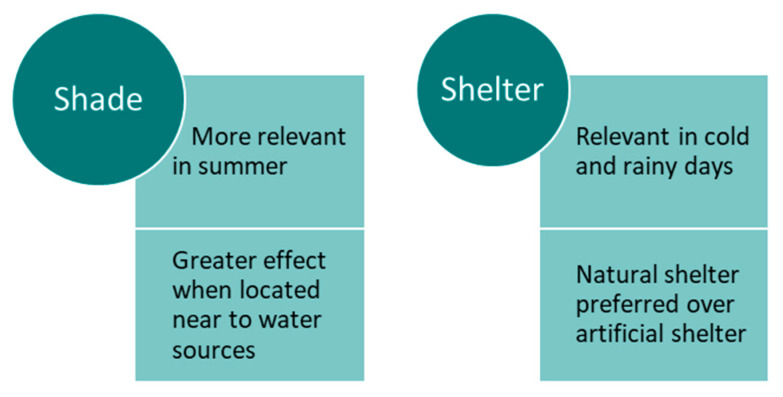
Main characteristics of the effect of shade and shelter on the distribution of cattle in the landscape.

Generally, all variables related to heat stress or heat-load, as well as cold weather, are associated with animal distribution in the grazing area, the use of the features of the field (shade, shelter, water sources, etc.) and their interaction with herbage attributes and air temperature [37]. For instance, in seasons when temperatures are not extreme, cows start grazing from nocturnal resting sites to subsequently graze away from the original grazing site, where then can either continue to an alternate site or return to the initial one, whereas in the summer, when temperatures during the day are extremely high, cows are forced to graze mainly in short round trips surrounding usual resting spots [37]. Interestingly, herd spread varies seasonally; it is greatest during summer and autumn and least during winter in semiarid rangelands [41]. Therefore, exploiting the interactions between these key factors influencing the activity patterns displayed by animals to cope with environmental conditions might stimulate livestock to use new areas of a paddock, e.g., providing shade, especially away from water sources [30]. The magnitude of these effects and the level of success on modifying animal distribution will depend upon the extent and duration of the exposure, and the biotype of the animal (locally adapted vs. introduced breeds), since it would modify the sensitivity of the animal to the environmental stimuli. The main characteristics of the effect that shade and shelter have on the distribution of cattle in the landscape are summarised in Figure 9.

### 4.3. Supplement Feed Location and Type

Animal distribution in pasture can be altered by placing rewards, such as supplements, e.g., feed or salt-licks, in locations where greater utilization is desired. These landscape interventions can modify livestock preference for particular zones and change habitat-use patterns [48] or distance travelled [49] to increase uniformity of foraging [50] and restore grasslands [51,52].

Low moisture block (LMB) and salt placed in low used locations far away from water in rangeland pastures (258 and 339-ha fields) grazed by cattle have proven to be effective in modifying cattle distribution in the field; both options result in a higher use of those areas of the field which were historically less used, and stimulate cattle to travel longer distances [53], particularly when the two supplements are placed together. Similarly, LMB placed in higher and steeper terrain seemed to be a more practical and successful strategy to improve the homogeneity of cattle grazing on rugged rangeland than traditional hand-feeding range cake fed on accessible areas [54].

**Figure 10:**
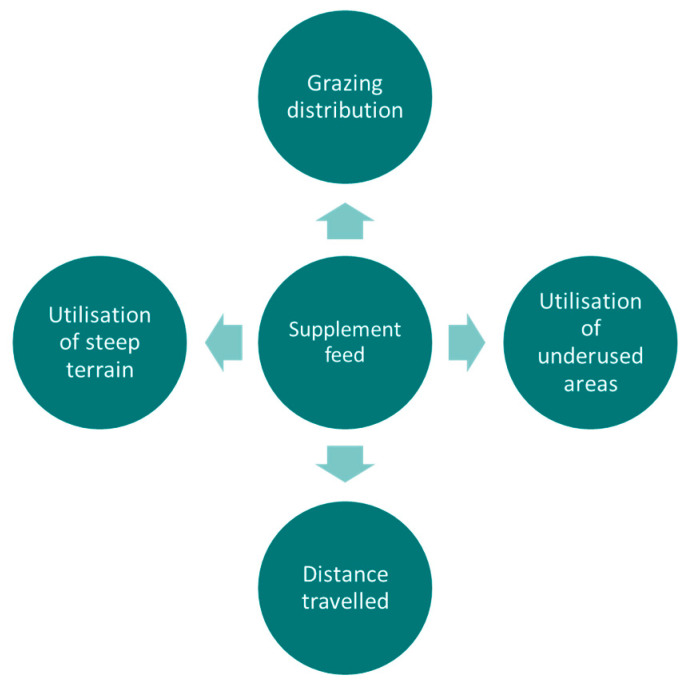
Summary diagram of the main variables of site use preference being affected by the location and number of supplemental feed points.

Interestingly, the provision of supplemental feed seems to interact with grazing management in their effect on cattle distribution. Cows at higher SR (0.55 cows/ha) graze further from supplement feeding stations than at moderate SR (0.33 cows/ha) grazing in a Mediterranean oak woodland [21]. On the other hand, cattle prefer grazing areas near salt supplements placement points regardless of the grazing method applied to large paddocks (i.e., RG or CS) in alpine grasslands [23], likely due to the similar SR achieved in the two systems. Figure 10 shows a summary of the main variables being affected by the location and number of supplemental feeds.

### 4.4. Vegetation Characteristics

The main determinant of animal distribution is cattle selectivity [34]. Generally, grazing herbivores chose plants and plant parts to optimise nutrient ingestion, as well as minimising energy cost and intake of detrimental chemical components [55]. It has been reported that a relevant proportion of cow movements (37%) is explained by the variation in resource availability [56]. Worldwide, the most important biotic factors which drive herbivore selectivity are linked to short plants at a wide range of scales and hierarchical levels [34,57], and the magnitude of other factors which effect the activity patterns depends on the specific context of the area [58].

Not surprisingly, the selection of certain vegetation areas is affected by the grazing management; when cattle are submitted to higher stocking densities (e.g., at higher SR or under RG) in heterogeneous mountain rangelands animals are forced to graze in less preferred areas and are not able to exert their selectivity for areas with higher forage pastoral value, thus the selection of flora ecological groups is more homogeneous [23,34]. In these habitats, cattle prefer lowland and upland grasslands, while avoiding conifer forests and cleared areas [59]. Additionally, the open grasslands and the tall herb community are the most visited vegetation groups, particularly early in the grazing season, whereas the shrubby areas are the least visited, which results in the cattle consuming forage of better and consistent quality (relatively high crude protein and rather low fibre contents) than the average [60]. Later in the season the spatial preferences follow a more random distribution [60], whilst in summer cows often graze around buildings and in regrowing zones that still have a grass-rich stratum, and follow old roads and paths between the grazing spots [61].

Cattle in heterogeneous subalpine pastures prefer to graze and rest on nutrient-rich vegetation than nutrient-poor vegetation, and grazing is less intense in patches of sparse forage [58]; dwarf shrub pastures are the least preferred vegetation, whereas the fertile pastures are the most preferred area [2]. This marked preference is also present in foothill ranges where free-ranging cattle show a marked preference for certain plant communities when grazing and resting [62]. Similarly, cattle grazing in lowland and sand-dune areas in China show greater foraging intensity in the lowlands where the herbage biomass and species diversity is higher [63]. Regarding the temporal variation of the vegetation preferences on grasslands, animals spend a shorter time foraging at the start of the grazing season, since the areas of plentiful palatable vegetation are closer. This leads to the early damage of vegetation due to overgrazing and, consequently, the animals reduce the time spent in those areas in order to visit new foraging areas [64]. It is likely the reduction in travelling distance required to reach the foraging areas becomes more determinant when the biomass of the preferred species decreases [64].

In relation to extensive grazing conditions such as rangelands, cattle show a similar pattern as in the grasslands; when experiencing high forage allowance conditions, they explore smaller areas of the pasture each day [45,46], travel shorter distances, and the herd is less spread [41]. In addition, they follow less sinuous pathways during nighttime hours, and show higher avoidance of woodland areas [22], with a higher occupancy rate on open grassland [65], particularly in cold winters (greater herbage availability in open grassland) [22]. In arid rangelands, cattle consistently select grazing areas with plant communities that have sufficient forage to meet their nutritional requirements and favour communities as resting areas that are dry and open (good visibility, a drier surface and less rocky soil) [66]. The herbaceous resource is not sufficient all year round, however, and when the herbaceous forage is scarce, free-ranging cows in Mediterranean oak (*Quercus* spp.) woodland spend most of their time in the dense woodland [21]. Additionally, the intense use of specific patches or vegetation types in extensive grazing cannot be reduced by using smaller paddocks, e.g., cattle in the smallest paddock can expend ~50% of their time in an area as small as 13% of the paddock [30]. Thus, despite SR and RG having been shown to be effective to improve the uniformity of cattle distribution in relation to vegetation types, that might not be the case for altering paddock size in extensive conditions. Regarding the nutritional value of pasture area preferentially grazed, cattle usually favour higher rather than average crude protein [28,67,68,69] (particularly in the evening [69]) and digestibility, and lower than average fibre [28]. However, for cattle grazing very extensive rangelands (9–57 km^2^), the palatable vegetation type that has the highest representation in the paddock is frequently the most grazed instead of those zones where palatable vegetation is most abundant, which is likely to reduce the time required to satisfy their energy needs in such a large areas [30].

The Normalized Difference Vegetation Index (NDVI) has been used as an indicator of herbage biomass and nutritional quality, i.e., the higher NDVI value the greater herbage mass and quality. Cattle show a strong preference for areas where NDVI is highest (≥0.5) [70], demonstrating that grazing patterns are influenced by the vegetation greenness [71] and forage quality [72,73]. Additionally, the seasonal variation of vegetation greenness also dictates the seasonal preference for grazing sites; in annual grassland pasture (~25 ha), low herbage nutritional value (and higher temperatures) in summer causes greatly concentrated grazing activity surrounding trees, whilst winter and early spring herbage of high nutritional value and low herbage mass motivate more widely dispersed grazing [37].

Nevertheless, as previously mentioned, the characteristics of the vegetation can interact with other landscape properties or features and modify the expected distribution of cattle; e.g., the animals can spend a significant proportion of time close to gates, which is the zone with lowest forage availability, resulting in the lowest NDVI values [73]. This site preference is explained by the hierarchy of behavioural drivers, i.e., the animal’s interest in the novelty of the gate and fences overturns the aversion of the lack of herbage in this zone [73].

A few studies have also analysed the effect of cattle and sheep co-grazing on pasture use. Even in a highly homogeneous sward, cattle and sheep have different innate spatial methods for exploring and exploiting the vegetation [74]. The feeding site selection of cattle and sheep appears to be primarily driven by forage-related (biotic) factors; cattle tend to select lower elevation sites, likely attributable to an energy-saving strategy by avoiding climbing hills [75], dominated by tall grass and mosaic vegetation types located further from water [38]. Additionally, cattle co-grazing on lowland grassland prefer heterogeneous patches, and interestingly, cattle and sheep graze among vegetation types complementarily of each other; where cattle preferentially graze in vegetation types located in wet places (ponds, sedge swamps, and wet meadows), these are the areas most avoided by sheep. In turn, low growing, unproductive plant communities on a hydromorphic site (dry, trampled, and nutrient-poor grasslands) are preferably grazed by sheep but utilised less by cattle [26].

**Figure 11:**
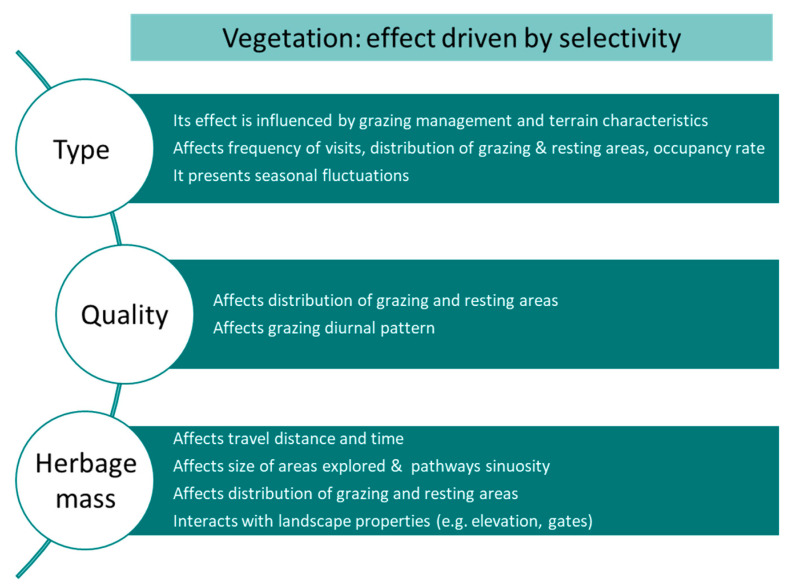
Main characteristics of the effect of vegetation on the distribution of cattle in the fields.

Forests may represent a very contrasting landscape compared to rangelands and grasslands when it comes to vegetation types affecting site selection by cattle. In boreal forests, cattle select the small patches of summer farm meadows and young forest regeneration stands of the bilberry-spruce forest [76], whilst selecting the most grass-rich site for grazing, and the flattest, most covered site for resting [39], which could be explained by the need to seek shelter from harassing insects [39,76]. The management of forests, such as logging and controlled fires, can also modify cattle’s use of the different areas. Cattle spend more time in uncut forests, attributed in part to the favourable forage quality, and avoid cleared areas and in-block haul roads [77]. Cattle selectively spend more time proportionally grazing recently burnt areas in mesic rangeland, especially during periods of rapid vegetation growth whenever it takes place [78]. Similarly, beef cows grazing mountainous, sagebrush (*Artemisia* spp.) steppe rangeland in spring regularly select for zones that had previously received low/moderate fire severity, and this preference is kept for at least five years [79]. Figure 11 shows a summary of the main characteristics of the effect of vegetation on site use preference of landscape by cattle.

### 4.5. Landscape—Topography

The environmentally determined differences in the cost of transport, driven by the variation in factors such as slope, substrate type, flora, current speed, or direction, is termed the “energy landscape”. The energy landscape may change in space and/or time, offering convincing energetic reasons for animals to adapt their movement approach correspondingly [80]. Thus, terrain slope is a critical landscape factor influencing cattle site use of the available area [58] and a significant predictor of grazing livestock distribution [28].

In rugged terrains, cattle establish least-effort routes (~5% less slope than the mean slope of the pasture) between distant points by selecting cross-slope routes [81], particularly in the wet-warm season [34], whereas cows grazing gentle topography and evenly distributed vegetation in rangelands rotate among feeding locations more often than cows grazing pastures with more rugged topography and more unevenly distributed vegetation [82]. Likewise, in the Alps’ grasslands cattle prefer areas with gentler terrain [52], whilst in Mediterranean grasslands beef cattle tend to prefer the flattest terrain sites [83]. When cattle are grazing in the undulating terrains of the lowland and sand-dune areas of inner Mongolia, their foraging density is greater in the lowlands [63]. Similarly, in heterogeneous subalpine pasture, the total activity (i.e., the overall presence of animals) of dairy cows tends to concentrate on the flat areas and around the buildings [2], and in boreal forests cattle select resting sites with a low incline [39]. However, in heterogeneous environments a daily pattern can be observed; during the day, cattle occupy sites with slopes lower than the average of the area [57], whereas at night they rest in aged (with little renovation), hilly areas [59], which aligns with the distribution in mesic sagebrush steppe; cattle select for higher mean elevation terrains, particularly in postfire years [79]. This highlights the complex interactions of the terrain characteristics and environments with the animal’s energy landscape. When considering their grazing patterns through the season, it was concluded that the grazing strategy of beef cattle is shaped by the interaction between the terrain, the distribution of the herbage mass and its nutritional composition; as herbage mass decreases throughout the growing season, the distribution of grazing becomes homogeneous and all terrain types are utilised [83].

Regarding soil conditions, cattle have the tendency to avoid black soils when they are wet because they become very soft, making walking difficult, whereas they use red and intermediate soils during the wet season (firmer condition) [30]. Within subtropical savanna and riparian areas, cows have shown a clear avoidance of areas of steep and stony terrains [84,85], while steers select sites associated with heavy clay and texture contrast soils, and avoid sodosols [20].

**Figure 12:**
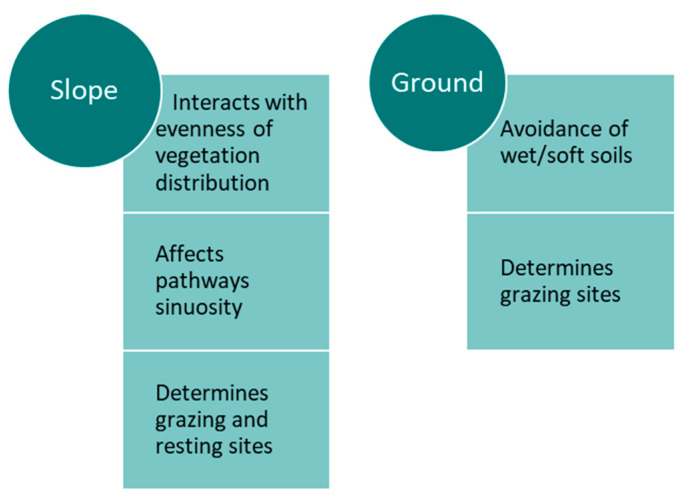
Main characteristics of the effect of slope and ground conditions on the distribution of cattle in the landscape.

When comparing animal species in mixed grazing, cattle tend to select lower elevation sites, whereas sheep tend to select higher elevation sites [38]. Energy landscape computations indicate that even light hills are significant energy barriers for heavy animals [75]. Along with slope, there may be other relevant motives for the large herbivores’ general evasion of climbing hills, such as overheating, risk of injury, lack of water or unavailability of forage [75]. For sheep, the preference for higher altitude feeding locations with augmented exposure to winds may be related to insect avoidance mechanisms [38]. Lastly, livestock distribution (cattle and sheep) is strongly affected by the location of the management facilities (reduction in travelling distance) [26]. The main characteristics of the effect that slope and ground conditions have on the distribution of cattle in the landscape are summarised in Figure 12.

## 5. Effect of Animal Factors and Social Interactions on Site Use Preference

The animal factors analysed in the studies identified between 2000 and 2020 covered individual traits and group characteristics. The individual traits were differences in phenotypes and genetics between animals, whereas the group characteristics included breed, physiological stage, age, previous experience, and social structure. The latter has been covered in a minor extent.

### 5.1. Phenotypes, Genetics and Breed

The management of livestock typically assumes that individual animals in a herd differ little in behavioural traits and respond similarly to management practices and actions [30]. However, both individual (phenotypes and/or genotypes) and group (breeds) diversity can impose different patterns of site use [42,43], and as such, individual differences in foraging behaviour may be useful for achieving more uniform grazing distribution. Furthermore, it has been shown that expressed animal individuality generates unique individual tracking patterns [13]. Therefore, these behavioural differences may be deliberately exploited to improve the management of grazing distribution [30].

Cows previously identified as phenotypically slow eaters of concentrate have a lower motivation to seek forage, a higher tendency to rest close to water or in sheltered areas than fast eaters [86]. However, cows of high and low feed efficiency do not differ in the daily distance travelled in steppe rangelands [44], and no relationship between liveweight gain and home range size or proportion of time spent on summer farm meadows was found in cows grazing on boreal forests [87]. Cows differing in their phenotypical habit to use hilly terrains graze different parts of the same pasture; “hill climbers” use rougher terrain and begin travelling to water about one hour later than cows previously classified as “bottom dwellers” [88], showing the degree of asynchrony in the motivation to eat or drink of different individuals [37]. However, other phenotypical traits (e.g., differences in pulmonary arterial pressure) between “hill climbers” or “bottom dwellers”, are not useful predictors of terrain preference within Angus cows adapted to high elevations [89].

Regarding genetic associations with grazing distribution, a chromosomal region (quantitative trait locus), associated with terrain-use indexes (accounting for slope use, elevation use, and distance travelled from water) has been identified [90,91]. The gene identified is involved in locomotion, motivation, and spatial memory. In addition, the genetic markers account for a relevant portion of the phenotypic variability in terrain use indexes. Therefore, grazing distribution can be inherited and offers a new approach to associated genetic variation in cattle grazing behaviour [90].

Variations among breeds are likely to be more readily identified than those among individual animals [30], and these differences can largely be explained by the different typical size or performance and the adaptation to local climates of the breeds. Large-frame mature cows (Beefmaster × Simford crossbred) are more active than small-frame mature cows (Baladi breed), foraging for more hours per day and walking longer distances [92]. Similarly, when located in less favourable areas, modern higher-yielding dairy cows (Holstein breed) prefer to graze in areas with more nutrient rich vegetation, whilst traditional lower-yielding cows (Swedish Mountain breed) prefer to graze in a less fertile area with low plant species diversity [93]. Besides, Swedish Mountain cows walk longer distances during grazing, spend less time in grass-dominated pasture and are dispersed over longer distances from other cows than Holstein cows [94]. This shows that traditional breeds are typically more adapted to use harsher environmental areas.

These differences between biotypes has also been observed between Zebu cattle (*Bos indicus*, Brahman breed) and a British breed (Angus) in desert areas [95]. Brahman cows travel longer daily distances than Angus cows and Brangus (Brahman × Angus) with no differences in average distance to water, whereas Angus cows maintain a more linear grazing route than Brangus or Brahman cows. Nevertheless, the difference in spatial movement patterns among biotypes do not suggest that there is any advantage in the use of locations far from water by any breed group in desert areas [95]. On the contrary, Raramuri Criollo cows (heritage breed) displays a larger home range size than those of Angus × Hereford throughout seasons with low herbage mass, but the home range sizes and spatial coverage of the herds converge during more productive seasons [96]. Additionally, Angus × Hereford have two-fold hotspots of use (locations with several visits of long duration), whereas Raramuri Criollo more strongly show the capacity to use nutritious forbs on open terrains despite summer heat, displaying a higher mobility per day and wider spatial dispersion throughout dry seasons [96]. On the other hand, when herbage mass is high and more evenly distributed through the landscape, animal foraging patterns are similar for Mexican Criollo (heritage breed) and Angus cows [97]. However, when herbage mass is low and nonevenly distributed, heritage animals forage through a much larger spatial coverage whereas their domestic equivalents remain very near to permanent water points.

### 5.2. Previous Experience and Physiological Stage

Reallocating cattle to a new environment can influence their site use patterns. Experienced cattle are more likely to use areas farther from water and higher in elevation, and older cattle graze closer to supplement locations [49]. In desert conditions, Brangus cows originating from a humid-subtropical environment use less area and stay closer to water than cows born and raised in the desert in their first winter in the new environment [47]. Additionally, during drought conditions, introduced cows select diets with lower crude protein compared to cows born in the desert, but during late summer after abundant precipitation they select a diet with higher crude protein [47]. In less extreme environments, rangeland-raised *Bos indicus* heifers need 4–6 weeks to adapt from their previous native grassland environment to a new temperate agricultural conditions, with an initial lesser grazing activity level, and lower productivity, as they become familiar with the new environmental conditions [98]. Although there is a clear impact of previous experience on grazing patterns and diet selection, it is likely these effects are transient and their relative importance can be lower compared with more permanent effects such as breed or physiological stage.

The physiological stage of cows has also shown to affect their foraging patterns. Pregnant or nursing cows (PN), grazing a woodland–grass steppe mosaic, show highest woodland preference on the day before or immediately after calving date. When compared with nonpregnant–nonlactating cows, PN cows explore smaller areas and travel shorter distances [40]. However, both in rangeland (desert) and in woodlands (semiarid) non-nursing cows exhibit straighter travel paths and explore larger daily areas than their nursing counterparts, showing no differences in distance travelled and time spent close to water troughs [99]. In semidesert rangelands cows’ behaviour differs between pre- and postweaning periods; before weaning, foraging behaviour of cows is not concentrated around the water points, whilst the travel rate intensifies during postweaning and the spatial distribution of cows is different compared with that of preweaning. Following weaning, also, foraging is associated with particular zones interconnected by paths/trails where walking takes place and therefore there is an overall increase in walking [100]. The liveweight of the calves has also been shown to affect the movements of the cows; cows that wean heavier calves tend to walk longer distances during the daytime hours in the weeks immediately following calving, with increased night-time distances travelled by cows with lighter calves [22]. These findings show the marked effect of the cows’ physiological stage on their distribution and movement patterns across the paddocks, which are likely to exert their effect every year seasonally. This in turn might interact with the vegetation and the weather conditions, making the prediction of the outcome more complex.

### 5.3. Social Structure

**Figure 13:**
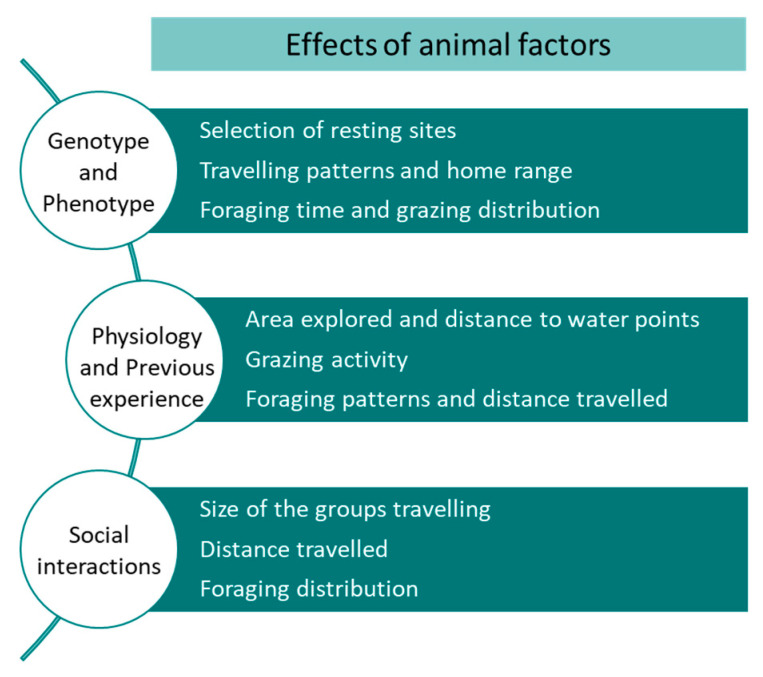
Main effects of animal factors on site use preference by cattle.

There are only a few studies that have used GPS tracking to account for the effect of social interactions on cattle grazing and movement patterns. Despite the fact that individual cattle from the same herd behave quite similarly to each other, it is common to find subgroups of animals that operate independently [101]. Forage availability and thermoregulatory needs influence the distance between associated subgroup members. When forage is abundant, herds travel in larger groups, whilst when it is scarce, herds fragment into subgroups, behaving more independently [102]. The association pattern shaping herd membership reveals that animals devote 70% of their time within 200 m of each other and dominance ranking does not seem to affect association membership or ranking within the herd [103]. Hence, foraging and short-distance travelling patterns by female beef cattle are not guided by any specific individual, but tend to be affected by a graded type of leadership; that is, the more dominant a cow is, the stronger the effect it may have on the movements of the herd [104]. This may have implications on the subgroup’s movements and use of resources, since cattle tend to establish home ranges (preferred portions of large pastures), proving quite persistent, even under adverse conditions [24]. It is clear that more research is needed to understand the effects of social interactions on cattle grazing patterns [88] and GPS is a valuable tool for this purpose. A summary of the main effects of animal factors on site use preference are shown in Figure 13.

## 6. Integrating GPS and Bio-Loggers Data

As mentioned previously, tracking data can be enhanced by combining location information with a corresponding activity assigned to it. This allows mapping the different activities (e.g., grazing, resting, ruminating, lying) to different areas within the pasture [16]. This can be achieved by using accelerometers and magnetometers along with the GPS devices [17]. Additionally, there are other types of bio-loggers that can be deployed in grazing ruminants to gain insights into their metabolism and its relationship with the landscape they utilise. There are many known relationships between basic physiological factors such as heart rate, body temperature and respiration and the state of health and metabolism of an animal [105]. By combining heart rate bio-loggers, motion sensors and GPS collars, activity costs and energy expenditure [106] or heat production [92] can be estimated. This approach has revealed that daily energy expenditure is influenced by numerous interdependent variables in addition to activity, including season, SR, herbage nutritional value, herbage mass, and the reproductive stage of the cow [106]. It has also shown that heat production of large- and small-frame cows on hilly pastures differs between groups throughout the four seasons [92]. Another example of the use of bio-loggers to complement the GPS data is the assessment of water intake behaviour. The amount of water drunk and its timing can be monitored by dosing reticulorumen temperature telemetry transmitters (“rumen boluses”) and relating drinking behaviour with site characteristics [107]. Temperature telemetry systems, either implantable or ingestible transmitters, and respiration rate sensors can also be deployed to examine the effects of heat stress [108] in relation to the surrounding environment (e.g., air temperature and humidity, solar radiation, use of shade by cattle) [36].

All these sources of information can be collated and processed following a precision livestock farming (PLF) approach to make informed decisions about farm management. Precision livestock farming is centred on the animal component and makes use of the heterogeneity in space and among individual animals towards more sustainable production systems. In this regard, a precision grazing system requires the definition of the variables to be measured, and particular inputs to create a catalogue of management actions relevant for these production systems [12]. With the progress made on the development of GPS devices and bio-loggers during the last two decades with regard to data transmission and processing, as well as with battery lifespan and size of the devices, it seems that the adoption of the PLF approach will increase in the coming years and the rate of adoption by farmers will depend more on the cost–benefit analysis rather than in the practicalities of its implementation and use.

## 7. Conclusions

Numerous factors influence the distribution of grazing cattle on pasture, namely: herbage mass characteristics and distribution; type and location of shade, water point, and supplemental feed; pasture and soil type, terrain incline and properties. Additionally, interventions such as alterations of the paddock shape and size, fence design, and grazing methods have the potential to influence cattle grazing patterns. Most of the studies have been carried out in rangelands/extensive pastures, in different zones of the world, but mainly in the US, with diverse weather and environmental conditions, livestock management and periods of time, number and proportion of cattle tracked, and techniques applied. The herd behaviour of the cattle grazing in small paddocks may exhibit different patterns than those in extensive rangeland grazing systems. Thus, there is potential to undertake research in more intensive grazing systems since the spatial pattern of cattle may vary with scale. Nevertheless, this varied repertory of studies is expanding our knowledge of the interactions in complex grazing systems, which can lead to new ideas for future research by incorporating the most useful and feasible findings and adapting the methodologies to the resources available and the research objectives, which will be climate, region and production system specific.

Applying the information provided in this review to intensive pasture grazing systems offers a rich potential to improve the productivity, sustainability, profitability, and animal welfare of beef farming operations. For instance, the relative amount of time animals spend displaying different behaviours throughout the grazing area could help to identify those areas that are overused (grazing hotspots) or resting sites and underused areas. This could inform the development of management strategies to modify cattle distribution in the landscapes, such as decreasing overgrazing and nutrient accumulation limited to small areas in resting sites through strategic location of water, shade, salt, and mineral points. The use of GPS devices complemented with bio-loggers (e.g., physiological variables, behaviour), along with appropriate software to interpret the data and generate information, may represent a relevant tool for precision livestock farming in its advancement towards more sustainable production systems.

## Supplementary Materials

The following are available online at https://www.mdpi.com/article/10.3390/s21082696/s1, Table S1: List of studies included and their main characteristics.
