# Supplementary material for: Factors Affecting Site Use Preference of Grazing Cattle Studied from 2000 to 2020 through GPS Tracking: A Review
Source: Sensors (Basel). 2021 Apr 11;21(8):2696. doi: 10.3390/s21082696 (PMC8069350; doi:10.3390/s21082696)
Supplement: Supplementary file 1 [file sensors-21-02696-s001.pdf]

Table S1. Summary of the main characteristics of the 84 studies published between 2000 and 2020 on the use of GPS tracking on cattle to determine the factors affecting their site use preference on grazing systems.

Reference	Years of study		Agroecosystem			Area	Herd and GPS collars					Factors assessed																				Response variables (only related to distribution)														
	Start	End	System type	Climate	Location	Average area (ha)	Average herd size	Average number of animals tracked	% Animals tracked	Animal type	Frequency GPS (s/signal)	Breed/biotype/frame	Physiological status/age	Phenotypic/genotypic traits	Previous experience	Social dominance	Topography/slope/elevation/insolation/visibility	Ground conditions: cover of rocks, soil moisture	Supplement: distance from/type	Water: distance to/elevation of	Distance to fence/shed/cattle tracks/roads	Shade: distribution/distance to/shelter type	Pasture size/herd size/stocking rate	Vegetation: units/groups, composition, heterogeneity, sparse forage	Trees: density, canopy cover, cutting age, burned area	Herbage quality	Herbage availability	Landscape/vegetation greenness (NDVI)	Grazing management, stocking intensity, stocking date, herder presence	Day vs Night	Environment: temperature, atmospheric pressure, weather, season	Travel (distance/intensity/rate/path/area covered)	Time foraging/grazing	Foraging/grazing site or path; home range; area explored	Resource/habitat selection; species preference	Ruminating or resting area/intensity	Forage utilisation/grazing intensity	Elevation/slope of terrain used	Distance to water/time within water area	Distance to supplement/time spent	Shade (distance to/use of/use of shelter)	Distance to roads/cattle tracks/fences	Tortuosity/crookedness of grazing pathways	Dispersion-aggregation pattern/stocking density	Social association	Frequency of visits
Aharoni et al. (2013)	2006	2007	Hilly grassland	Mediterranean	Israel	28	16.5	16.5	100	Beef cows	300	x																				x	x													
Anderson et al. (2012)	2009	2011	Rangeland	Semi-desert	USA	433	21	8.5	58.3	Beef cows	1		x																			x		x												
Augustine and Derner (2014)	2007	2011	Rangeland	Semi- arid	USA	65		3.0		Steers	300						x									x									x												
Bailey et al. (2001)	1998	1999	Foothills rangeland	Semi- arid	USA	288	159	81.0	50.9	Beef cows	600						x		x	x																	x									
Bailey et al. (2004)	1998	1998	Foothills rangeland	Semi- arid	USA	337	160	9.0	5.6	Beef cows	600, 1200			x																		x						x	x				x			
Bailey et al. (2008)	2004	2004	Foothills rangeland	Semi- arid	USA	294	98	9.0	9.2	Beef cows	600								x													x						x	x	x						
Bailey et al. (2010)	2005	2006	Rangeland	Desert	USA	2210	7	1.0	14.3	Beef cows	600				x																	x			x					x						
Bailey et al. (2016)	2013	2014	Rangeland	Continental humid	USA	1210	300	20.5	6.8	Beef cows	600			x																								x	x							
Bailey and Jensen (2008)	2002	2002	Foothills rangeland	Semi- arid	USA	294	80	22.5	28.1	Beef cows	600								x													x						x	x	x						
Bailey and Welling (2007) Study 1	2000	2000	Foothills rangeland	Semi-arid	USA	147	214	10.0	4.7	Beef cows	600						x		x																					x						
Bailey and Welling (2007) Study 2	2001	2002	Foothills rangeland	Semi-arid	USA	213	167	25.0	15.1	Beef cows	600								x																											
Bailey, Lunt et al. (2015)	2009	2013	Rangeland, mountainous and/or extensive pasture	Semi- arid	USA	2648	113	16.7	21.2	Beef cows	600			x																								x	x							
Bailey, Stephenson et al. (2015)	2010	2012	Rangeland, mountainous and/or extensive pasture	Semi- arid	USA	2318	344	15.4	10.1	Beef cows	600, 900						x							x										x												
Bear et al. (2012) Study 1	2007	2009	Rangeland	Continental humid	USA	58	42	2.5	7.6	Beef cows	600											x	x	x							x								x							
Bear et al. (2012) Study 2	2009	2009	Rangeland	Continental humid	USA	11	17	2.5	16.2	Beef heifers	600											x																	x							
Browning et al. (2018)	2008	2008	Rangeland	Desert	USA	1525	10	7.3	70.1	Beef cows	300																	x				x	x	x									x			
Byers et al. (2005)	2001	2001	Riparian pasture	Subtropical	USA	16	20	3.0	15.0	Beef cows	300											x																	x							
Cheleuitte-Nieves et al. (2018)	2008	2009	Rangeland	Semi-arid	USA	100	10	10.0	100	Beef cows	300					x															x													x		
Cheleuitte-Nieves et al. (2020)	2007	2008	Rangeland	Semi-arid	USA	457	31	11.0	35	Beef cows	300								x	x		x						x			x													x		
Clark et al. (2014)	2003	2009	Rangeland	Semi-arid	USA	324	800	10.0	1.3	Beef cows	600														x										x											
Cooper et al. (2008)	2005	2006	Rangeland	Semi-arid	USA	1651	60	9.0	15.0	Beef cows	300									x															x				x							
Diaz Falu et al. (2014)	2009	2011	Grassland	Sub-tropical	Argentina	128	64	7.0	10.9	Beef cows & ewes	600						x			x		x		x										x												
Eikelboom et al. (2020)	2017	2017		Temperate oceanic	Netherland	1	8	8.0	100.0	Dairy cows	1																x					x	x													x
Ganskopp (2001)	1999	1999	Rangeland	Arid	USA	842	40	2.0	5.0	Beef cows	600								x	x												x	x		x				x	x						
Ganskopp and Bohnert (2009)	2004	2004	Rangeland	Arid	USA	847	20	4.0	20.0	Beef cows	300						x			x						x																				x
Gonzalez et al. (2014)	2013	2014	Open woodland	Tropical savannah	Australia	15	30	20.0	66.7	Steers	10																									x	x									
Gou et al. (2020)	2018	2018	Lowland grassland and dunes areas	Semi-arid	China	20	13	13.0	100.0		50						x							x		x	x										x									
Haan et al. (2010)	2005	2007	Grassland with trees	Continental humid	USA	12	15	1.0	6.7	Beef cows	600									x									x		x				x				x							
Halasz et al. (2016)	2010	2013	Rangeland	Continental humid	Hungary	1191	200	10.0	5.0	Beef cows																	x				x	x	x													
Henkin et al. (2012)	2002	2003	Hilly grassland	Mediterranean	Israel	28	23	3.8	18.0	Beef cows	300						x	x					x											x												
Hessle et al. (2008)	2004	2004	Semi-natural grassland	Mediterranean	Sweden	18	14	12.0	85.7	Beef heifers	15	x						x													x	x	x													
Homburger et al. (2015)	2011	2012	Upland grassland	Alpine	Switzerland	84	57	2.8	5.7	Beef cows and dairy cows	20						x			x		x	x	x		x						x				x	x							x		
Hunt et al. (2007)	2003	2006	Rangeland	Semi-arid	Australia	3333	314	3.0	2.1	Beef cows	3600									x			x			x	x							x												
Johnson, Clark et al. (2016)	2008	2012	Rangeland, mountainous	Continental Mediterranean	USA	14663		10.0		Beef cows	300									x														x					x							
Johnson, Larson et al. (2016)	2008	2012	Rangeland, mountainous	Continental Mediterranean	USA	14663		10.0		Beef cows	300									x														x					x							
Kaucner et al. (2013)	2007	2008	Grassland	Temperate oceanic	Australia	25	14	6.0	57.9	Beef cows	600									x		x																	x		x					
Kaufmann, Bork, Alexander et al. (2013)	2008	2009	Foothill open woodlands	Continental subarctic	Canada	6	4	4.0	100	Beef cows	300									x			x		x		x								x											x
Kaufmann, Bork, Blenis et al. (2013)	2008	2009	Rangeland and forest	Continental subarctic	Canada	745	311	11.0	3.5	Beef cows	600						x			x						x	x								x		x					x				x
Koch et al. (2018)	2011	2011	Alpine grassland	Alpine	Switzerland		47	3.0	6.4	Dairy cows	20													x																						x
Larson et al. (2017)	2009	2010	Riparian pasture	Mediterranean	USA	57		10.0		Beef cows	1									x				x									x			x			x							
Larson-Praplan et al. (2015)	2001	2003	Foothills rangeland	Mediterranean	USA	23	20	6.0	30.0	Beef cows	300														x	x					x			x							x		x			
Manning et al. (2017)	2015	2015	Grassland	Temperate Oceanic	Australia	9	20	10.0	50.0	Beef cows	300																	x						x												
Meisser et al. (2014)	2006	2007	Alpine grassland/mountain pasture	Alpine	Switzerland	3	18	4.0	22.2	Beef cows	1, 10													x	x	x									x											
Nyamuryekung’e et al. (2020) [site 1]	2015	2016	Rangeland	Desert	USA	4355	33	11.5	35.4	Beef cows and calves	300			x																		x		x					x				x			
Nyamuryekung’e et al. (2020) [site 2]	2015	2016	Woodland/Grassland	Semi-arid	USA	623	78	11.5	14.8	Beef cows and calves				x																		x		x					x				x			
Orr et al. (2012)	2005	2005	Semi-natural grassland	Temperate oceanic	England	2	4	2.0	50.0	Beef heifers	1												x											x												
Pandey et al. (2009)	2001	2003	Rangeland, mosaic (open grassland, forest, wetlands)	Subtropical humid	USA	26	23	2.8	12.0	Beef cows	900																				x								x							
Peinetti et al. (2011)	2005	2005	Rangeland	Desert	USA	2425	12	12.0	100	Beef cows	300	x					x			x				x			x				x			x												
Pierce et al. (2020)	2010	2018	Rangeland		USA					Beef cows	600			x																								x	x							
Pittarello et al. (2015)	2013	2014	Alpine grassland	Alpine	Italy	44	113	14.0	12.5	Beef heifers and cows	900						x		x	x				x		x												x							x
Probo et al. (2013)	2010	2010	Alpine grassland	Alpine	Italy	365	135	7.0	5.3	Beef heifers and cows	900								x															x						x						
Probo et al. (2014)	2010	2012	Alpine grassland	Alpine	Italy	98	130	7.0	5.4	Beef heifers and cows	900						x		x	x				x	x	x	x		x				x	x					x	x				x		
Putfarken et al. (2008)	2003	2003	Lowland grassland	Temperate oceanic	Germany	180	74	3.0	4.1	Beef cows and sheep	300						x			x	x			x											x											
Riaboff et al. (2020)	2018	2018	Grassland	Temperate oceanic	France	2	71	26.0	36.6	Dairy cows	1						x	x						x									x			x										x
Rinella et al. (2011)	1990	2007	Rangeland, mountainous	Mediterranean	USA	2373	500	39.5	7.9	Beef cows																			x		x			x												
Rubio et al. (2008)	2004	2004	Rangeland, cleared woodland	Semi-arid	USA	183	87	4.0	4.6	Beef cows	300		x																		x	x		x	x											
Russell et al. (2012)	2008	2009	Rangeland	Desert	USA	2168	7	2.0	28.6	Beef cows	60	x																				x						x	x			x				
Saether et al. (2006)	2002	2002	Semi-natural mountain grassland	Transitional	Norway			1.0		Dairy cows				x																		x			x											

Sant'Anna et al. (2015)	2007	2007	Semi-natural grassland	Continental humid	Sweden	64	26	1.0	3.8	Beef cows	300						x			x					x										x				x			x				x
Šárová et al. (2010)	2005	2005	Grassland	Continental	Czech Republic	2.3	15	15.0	100.0	Beef cows	1					x																x		x		x										
Sather et al. (2013)	2009	2010	Grassland	Continental humid and Subtropical humid	USA	21	28	3.0	13.9	Beef cows	3600													x																						x
Sawalhah et al. (2014)	2004	2007	Woodland/Grassland	Semi-arid	USA	146	50	13.0	54.9	Beef cows	300						x			x	x				x		x				x			x												
Sawalhah et al. (2016)	2004	2007	Woodland/Grassland	Semi-arid	USA	146	50	13.0	54.9	Beef cows	300																			x	x	x		x	x								x			
Schlecht et al. (2004)	1998	1998	Livestock-crop rotation	Semi-arid	Nigeria			14.0		Beef cows	10																		x		x	x	x													
Schlecht et al. (2006)	1997	1999	Livestock-crop rotation	Semi-arid	Nigeria	29800		12.0		Beef cows, sheep and goats	10																		x		x	x	x													
Schoenbaum et al. (2017)	2007	2009	Oak woodland	Mediterranean	Israel	53	24	8.0	37.8	Beef cows	300						x							x					x		x		x		x				x	x		x				
Schwarte et al. (2011)	2008	2009	Grassland with trees	Continental humid	USA	12	15	1.0	6.7	Beef cows	600									x									x		x				x				x							
Sickel et al. (2004)	2000	2000	Semi-natural mountain grassland	Transitional	Norway		14	1.0	7.2	Dairy cows																						x		x	x							x				
Spedener et al. (2019)	2017	2017	South-boreal forest (coniferous)	Continental	Norway	13500	740	16.0	2.2	Beef cows and sheep	300						x							x	x									x	x	x										
Spiegal et al. (2019)	2008	2008	Rangeland	Desert	USA	1535	11	10.1	92.8	Beef cows	300	x												x							x			x	x											
Sprinkle et al. (2020)	2015	2015	Rangeland	Continental	USA	323	30	10.0	33.3	Beef cows	120			x																		x														
Stephenson and Bailey (2017)	2010	2012	Rangeland	Desert, semi-arid	USA	2318	130	14.8	17.0	Beef cows	600, 900						x			x			x																							
Thomas et al. (2011)	2008	2008	Rangeland	Mediterranean	Australia	41	109	6.0	5.6	Beef heifers	300	x			x																	x		x												
Thompson et al. (2015)	2004	2005	Foothill grassland (Stipa-poa zone)	Continental	Canada	65	30	7.0	23.3	Beef cows	300													x								x		x	x	x										
Tofastrud et al. (2019)	2015	2017	South-boreal forest (coniferous)	Continental	Norway	12500		52.0		Beef cows	300, 600						x				x		x	x	x									x	x											
Tofastrud et al. (2020)	2015	2017	South-boreal forest (coniferous)	Continental	Norway	12500	336	53.0	15.8	Beef cows	300			x																				x	x											
Tomkins et al. (2009)	2004	2006	Rangeland	Subtropical Savanna	Australia	105	27	3.0	12.6	Beef Steers	1800, 3600												x	x			x	x				x		x	x											x
Tomkins and O'Reagain (2007)	2005	2006	Rangeland	Subtropical Savanna	Australia	1530	183	12.0	6.6	Beef cows	1800						x							x			x					x		x	x				x							
Turner et al. (2000)	1997	1998	Pasture	Subtropical humid	USA	6	24	7.0	29.2	Beef cows and steers	300																				x		x		x		x									x
Van Laer, Ampe et al. (2015)	2011	2014	Natural reserve	Temperate oceanic	Belgium	54	10	1.0	11.6	Beef cows and oxes	900, 1800											x		x							x				x						x					
Van Laer, Moons et al. (2015)	2012	2013	Natural reserve	Temperate oceanic	Belgium	51	10	1.0	11.6	Beef cows and oxes	1800											x		x							x				x						x					
Watanabe et al. (2010)	2005	2005	Pasture and woodland	Continental humid	Japan	3	10	2.0	20.0	Beef cows	600						x									x	x							x		x										
Wesley et al. (2012)	2006	2007	Rangeland, grassland and woodland	Semi- arid	USA	146	18	18.0	100	Beef cows	300																					x		x					x							
Wyffels et al. (2019)	2015	2017	Rangeland	Semi-arid	USA	68	50	7.0	14.0	Beef heifers	300						x		x							x	x				x		x		x											
Wyffels et al. (2020)	2016	2017	Rangeland	Desert, semi-arid	USA	329	287	30.0	10.5	Beef cows	900		x	x			x		x												x	x			x			x	x	x						
Zengeya et al. (2013)	2008	2009	Communal land and conservation (savanna)	Semi-arid	Zimbabwe			1.0		Beef cows	3600															x		x						x												
Zengeya et al. (2015)	2009	2010	Communal land	Semi-arid	Zimbabwe			1.0		Beef cows	300																	x	x			x	x	x												

List of references

Aharoni Y., Dolev A., Henkin Z., Yehuda Y., Ezra A., Ungar E. D., Shabtay A., and Brosh A. (2013). Foraging behavior of two cattle breeds, a whole-year study: I. Heat production, activity, and energy costs. *J. Anim. Sci.* **91**, 1381–1390. doi:10.2527/jas.2012-5400

Anderson D. M., Winters C., Estell R. E., Fredrickson E. L., Doniec M., Detweiler C., Rus D., James D., and Nolen B. (2012). Characterising the spatial and temporal activities of free-ranging cows from GPS data. *Rangel. J.* **34**, 149–161. doi:10.1071/RJ11062

Augustine D. J., and Derner J. D. (2014). Controls over the strength and timing of fire-grazer interactions in a semi-arid rangeland. *J. Appl. Ecol.* **51**, 242–250. doi:10.1111/1365-2664.12186

Bailey D. W., and Jensen D. (2008). Method of supplementation may affect cattle grazing patterns. *Rangel. Ecol. Manag.* **61**, 131–135. doi:10.2111/06-167.1

Bailey D. W., Keil M. R., and Rittenhouse L. R. (2004). Research observation: Daily movement patterns of hill climbing and bottom dwelling cows. *J. Range Manag.* **57**, 20–28. doi:10.2307/4003950

Bailey D. W., Lunt S., Lipka A., Thomas M. G., Medrano J. F., Canovas A., Rincon G., Stephenson M. B., and Jensen D. (2015). Genetic Influences on Cattle Grazing Distribution: Association of Genetic Markers with Terrain Use in Cattle. *Rangel. Ecol. Manag.* **68**, 142–149. doi:10.1016/j.rama.2015.02.001

Bailey D. W., Stephenson M. B., and Pittarello M. (2015). Effect of terrain heterogeneity on feeding site selection and livestock movement patterns. *Anim. Prod. Sci.* **55**, 298–308. doi:10.1071/AN14462

Bailey D. W., Thomas M. G., Holt T. N., Stephenson M. B., Enns R. M., and Speidel S. E. (2016). Relationship of pulmonary arterial pressure and terrain use of Angus cows grazing high-altitude foothill rangelands. *Livest. Sci.* **190**, 76–80. doi:10.1016/j.livsci.2016.06.003

Bailey D. W., Thomas M. G., Walker J. W., Witmore B. K., and Tolleson D. (2010). Effect of Previous Experience on Grazing Patterns and Diet Selection of Brangus Cows in the Chihuahuan Desert. *Rangel. Ecol. Manag.* **63**, 223–232. doi:10.2111/08-235.1

Bailey D. W., Vanwagoner H. C., Weinmeister R., and Jensen D. (2008). Comparison of low-moisture blocks and salt for manipulating grazing patterns of beef cows. *J. Anim. Sci.* **86**, 1271–1277. doi:10.2527/jas.2007-0578

Bailey D. W., and Welling G. R. (2007). Evaluation of low-moisture blocks and conventional dry mixes for supplementing minerals and modifying cattle grazing patterns. *Rangel. Ecol. Manag.* **60**, 54–64. doi:10.2111/05-138R1.1

Bailey D. W., Welling G. R., and Miller E. T. (2001). Cattle use of foothills rangeland near dehydrated molasses supplement. *J. RANGE Manag.* **54**, 338–347. doi:10.2307/4003101

Bear D. A., Russell J. R., and Morrical D. G. (2012). Physical characteristics, shade distribution, and tall fescue effects on cow temporal/spatial distribution in midwestern pastures. *Rangel. Ecol. Manag.* **65**, 401–408. doi:10.2111/REM-D-11-00072.1

Browning D. M., Spiegal S., Estell R. E., Cibils A. F., and Peinetti R. H. (2018). Integrating space and time: a case for phenological context in grazing studies and management. *Front. Agric. Sci. Eng.* **5**, 44–56. doi:10.15302/J-FASE-2017193

Byers H. L., Cabrera M. L., Matthews M. K., Franklin D. H., Andrae J. G., Radcliffe D. E., McCann M. A., Kuykendall H. A., Hoveland C. S., and Calvert V. H. (2005). Phosphorus, sediment, and Escherichia coli loads in unfenced streams of the Georgia Piedmont, USA. *J. Environ. Qual.* **34**, 2293–2300. doi:10.2134/jeq2004.0335

Cheleuitte-Nieves C. T., Perotto-Baldivieso H. L., Wu X. Ben, and Cooper S. M. (2018). Association patterns reveal dispersal-aggregation dynamics among cattle in a South Texas Rangeland, USA. *Ecol. Process.* **7**,. doi:10.1186/s13717-018-0141-9

Cheleuitte-Nieves C., Perotto-Baldivieso H. L., Wu X. Ben, and Cooper S. M. (2020). Environmental and landscape influences on the spatial and temporal distribution of a cattle herd in a South Texas rangeland. *Ecol. Process.* **9**,. doi:10.1186/s13717-020-00245-6

Clark P. E., Lee J., Ko K., Nielson R. M., Johnson D. E., Ganskopp D. C., Chigbrow J., Pierson F. B., and Hardegree S. P. (2014). Prescribed fire effects on resource selection by cattle in mesic sagebrush steppe. Part 1: Spring grazing. *J. Arid Environ.* **100**, 78–88. doi:10.1016/j.jaridenv.2013.10.012

Cooper S. M., Perotto-Baldivieso H. L., Owens M. K., Meek M. G., and Figueroa-Pagan M. (2008). Distribution and interaction of white-tailed deer and cattle in a semi-arid grazing system. *Agric. Ecosyst. Environ.* **127**, 85–92. doi:10.1016/j.agee.2008.03.004

Diaz Falu E. M., Angel Brizuela M., Silvia Cid M., Francisco Cibils A., Gabriela Cendoya M., and Bendersky D. (2014). Daily feeding site selection of cattle and sheep co-grazing a heterogeneous subtropical grassland. *Livest. Sci.* **161**, 147–157. doi:10.1016/j.livsci.2013.11.010

Eikelboom J. A. J., de Knegt H. J., Klaver M., van Langevelde F., van der Wal T., and Prins H. H. T. (2020). Inferring an animal's environment through biologging: quantifying the environmental influence on animal movement. *Mov. Ecol.* **8**, 1–18. doi:10.1186/s40462-020-00228-4

Ganskopp D. (2001). Manipulating cattle distribution with salt and water in large arid-land pastures: a GPS/GIS assessment. *Appl. Anim. Behav. Sci.* **73**, 251–262. doi:10.1016/S0168-1591(01)00148-4

Ganskopp D. C., and Bohnert D. W. (2009). Landscape nutritional patterns and cattle distribution in rangeland pastures. *Appl. Anim. Behav. Sci.* **116**, 110–119. doi:10.1016/j.applanim.2008.10.006

Gonzalez L. A., Bishop-Hurley G., Henry D., Charmley E., González L. A., Bishop-Hurley G., Henry D., and Charmley E. (2014). Wireless sensor networks to study, monitor and manage cattle in grazing systems. *Anim. Prod. Sci.* **54**, 1687–1693. doi:10.1071/AN14368

Gou X., Tsunekawa A., Tsubo M., Peng F., Sun J., Li Y., Zhao X., and Lian J. (2020). Seasonal dynamics of cattle grazing behaviors on contrasting landforms of a fenced ranch in northern China. *Sci. Total Environ.* **749**, 141613. doi:10.1016/j.scitotenv.2020.141613

Haan M. M., Russell J. R., Davis J. D., and Morrical D. G. (2010). Grazing Management and Microclimate Effects on Cattle Distribution Relative to a Cool Season Pasture Stream. *Rangel. Ecol. Manag.* **63**, 572–580. doi:10.2111/REM-D-09-00045.1

Halasz A., Nagy G., Tasi J., Bajnok M., and Mikone J. E. (2016). Weather regulated cattle behaviour on rangeland. *Appl. Ecol. Environ. Res.* **14**, 149–158. doi:10.15666/aeer/1404_149158

Henkin Z., Ungar E. D., and Dolev A. (2012). Foraging behaviour of beef cattle in the hilly terrain of a Mediterranean grassland. *Rangel. J.* **34**, 163–172. doi:10.1071/RJ11096

Hessle A., Rutter M., and Wallin K. (2008). Effect of breed, season and pasture moisture gradient on foraging behaviour in cattle on semi-natural grasslands. *Appl. Anim. Behav. Sci.* **111**, 108–119. doi:10.1016/j.applanim.2007.05.017

Homburger H., Lüscher A., Scherer-Lorenzen M., and Schneider M. K. (2015). Patterns of livestock activity on heterogeneous subalpine pastures reveal distinct responses to spatial autocorrelation, environment and management. *Mov. Ecol.* **3**, 1–15. doi:10.1186/s40462-015-0053-6

Hunt L. P., Petty S., Cowley R., Fisher A., Ash A. J., and MacDonald N. (2007). Factors affecting the management of cattle grazing distribution in northern Australia: Preliminary observations on the effect of paddock size and water points. *Rangel. J.* **29**, 169–179. doi:10.1071/RJ07029

Johnson D. E., Clark P. E., Larson L. L., Wilson K. D., Louhaichi M., Freeburg T., and Williams J. (2016). Cattle use of off-stream water developments across a northeastern Oregon landscape. *J. SOIL WATER Conserv.* **71**, 494–502. doi:10.2489/jswc.71.6.494

Johnson D. E., Larson L. L., Wilson K. D., Clark P. E., Williams J., and Louhaichi M. (2016). Cattle use of perennial streams and associated riparian areas on a northeastern Oregon landscape. *J. SOIL WATER Conserv.* **71**, 484–493. doi:10.2489/jswc.71.6.484

Kaucner C. E., Whiffin V., Ray J., Gilmour M., Ashbolt N. J., Stuetz R., and Roser D. J. (2013). Can off-river water and shade provision reduce cattle intrusion into drinking water catchment riparian zones? *Agric. WATER Manag.* **130**, 69–78. doi:10.1016/j.agwat.2013.08.012

Kaufmann J., Bork E. W., Alexander M. J., and Blenis P. V (2013). Habitat selection by cattle in Foothill landscapes following variable harvest of aspen forest. *For. Ecol. Manage.* **306**, 15–22. doi:10.1016/j.foreco.2013.06.004

Kaufmann J., Bork E. W., Blenis P. V, and Alexander M. J. (2013). Cattle habitat selection and associated habitat characteristics under free-range grazing within heterogeneous Montane rangelands of Alberta. *Appl. Anim. Behav. Sci.* **146**, 1–10. doi:10.1016/j.applanim.2013.03.014

Koch B., Homburger H., Edwards P. J., and Schneider M. K. (2018). Phosphorus redistribution by dairy cattle on a heterogeneous subalpine pasture, quantified using GPS tracking. *Agric. Ecosyst. Environ.* **257**, 183–192. doi:10.1016/j.agee.2017.10.002

Van Laer E., Ampe B., Moons C., Sonck B., and Tuyttens F. A. M. (2015). Wintertime use of natural versus artificial shelter by cattle in nature reserves in temperate areas. *Appl. Anim. Behav. Sci.* **163**, 39–49. doi:10.1016/j.applanim.2014.12.004

Van Laer E., Moons C. P. H., Ampe B., Sonck B., Vangeyte J., and Tuyttens F. A. M. (2015). Summertime use of natural versus artificial shelter by cattle in nature reserves. *Anim. Welf.* **24**, 345–356. doi:10.7120/09627286.24.3.345

Larson-Praplan S., George M. R., Buckhouse J. C., and Laca E. A. (2015). Spatial and temporal domains of scale of grazing cattle. *Anim. Prod. Sci.* **55**, 284–297. doi:10.1071/AN14641

Larson L., Johnson D. E., Wilson M., Wilson K., Louhaichi M., and Williams J. (2017). Spatial occupancy patterns and activity of arid rangeland cattle grazing small riparian pastures. *Anim. Sci. J.* **88**, 553–558. doi:10.1111/asj.12670

Manning J. K., Cronin G. M., González L. A., Hall E. J. S., Merchant A., and Ingram L. J. (2017). The behavioural responses of beef cattle (Bos taurus) to declining pasture availability and the use of gnss technology to determine grazing preference. *Agric.* **7**, 1–12. doi:10.3390/agriculture7050045

Meisser M., Deléglise C., Freléchoux F., Chassot A., Jeangros B., and Mosimann E. (2014). Foraging behaviour and occupation pattern of beef cows on a heterogeneous pasture in the swiss alps. *Czech J. Anim. Sci.* **59**, 84–95. doi:10.17221/7232-cjas

Nyamuryekung'e S., Cibils A. F., Estell R. E., VanLeeuwen D., Steele C., Estrada O. R., Almeida F. A. R., González A. L., and Spiegal S. (2020). Do young calves influence movement patterns of nursing Raramuri Criollo cows on rangeland? *Rangel. Ecol. Manag.* **73**, 84–92. doi:10.1016/j.rama.2019.08.015

Orr R. J., Tozer K. N., Griffith B. A., Champion R. A., Cook J. E., and Rutter S. M. (2012). Foraging paths through vegetation patches for beef cattle in semi-natural pastures. *Appl. Anim. Behav. Sci.* **141**, 1–8. doi:10.1016/j.applanim.2012.07.003

Pandey V., Kiker G. A., Campbell K. L., Williams M. J., and Coleman S. W. (2009). GPS Monitoring of cattle location near water features in South Florida. *Appl. Eng. Agric.* **25**, 551–562. doi:doi:10.13031/2013.27465

Peinetti H. R., Fredrickson E. L., Peters D. P. C., Cibils A. F., Octavio Roacho-Estrada J., and Laliberte A. S. (2011). Foraging behavior of heritage versus recently introduced herbivores on desert landscapes of the American Southwest. *ECOSPHERE* **2**,. doi:10.1890/ES11-00021.1

Pierce C. F., Speidel S. E., Coleman S. J., Enns R. M., Bailey D. W., Medrano J. F., Cánovas A., Meiman P. J., Howery L. D., Mandeville W. F., and Thomas M. G. (2020). Genome-wide association studies of beef cow terrain-use traits using Bayesian multiple-SNP regression. *Livest. Sci.* **232**,. doi:10.1016/j.livsci.2019.103900

Pittarello M., Probo M., Lonati M., Bailey D. W., and Lombardi G. (2015). Effects of traditional salt placement and strategically placed mineral mix supplements oncattle distribution in the Western Italian Alps. *GRASS FORAGE Sci.* **71**, 529–539. doi:10.1111/gfs.12196

Probo M., Lonati M., Pittarello M., Bailey D. W., Garbarino M., Gorlier A., and Lombardi G. (2014). Implementation of a rotational grazing system with large paddocks changes the distribution of grazing cattle in the south-western Italian Alps. *Rangel. J.* **36**, 445–458. doi:10.1071/RJ14043

Probo M., Massolo A., Lonati M., Bailey D. W., Gorlier A., Maurino L., and Lombardi G. (2013). Use of mineral mix supplements to modify the grazing patterns by cattle for the restoration of sub-alpine and alpine shrub-encroached grasslands. *Rangel. J.* **35**, 85–93. doi:10.1071/RJ12108

Putfarken D., Dengler J., Lehmann S., and Härdtle W. (2008). Site use of grazing cattle and sheep in a large-scale pasture landscape: A GPS/GIS assessment. *Appl. Anim. Behav. Sci.* **111**, 54–67. doi:10.1016/j.applanim.2007.05.012

Riaboff L., Couvreur S., Madouasse A., Roig-Pons M., Aubin S., Massabie P., Chauvin A., Bédère N., and Plantier G. (2020). Use of predicted behavior from accelerometer data combined with GPS data to explore the relationship between dairy cow behavior and pasture characteristics. *Sensors (Switzerland)* **20**, 1–33. doi:10.3390/s20174741

Rinella M. J., Vavra M., Naylor B. J., and Boyd J. M. (2011). Estimating influence of stocking regimes on livestock grazing distributions. *Ecol. Modell.* **222**, 619–625. doi:10.1016/j.ecolmodel.2010.10.004

Rubio C. M. B., Cibils A. F., Endecott R. L., Petersen M. K., and Boykin K. G. (2008). Pinon-juniper woodland use by cattle in relation to weather and animal reproductive state. *Rangel. Ecol. Manag.* **61**, 394–404.

Russell M. L., Bailey D. W., Thomas M. G., and Witmore B. K. (2012). Grazing Distribution and Diet Quality of Angus, Brangus, and Brahman Cows in the Chihuahuan Desert. *Rangel. Ecol. Manag.* **65**, 371–381. doi:10.2111/REM-D-11-00042.1

Saether N. H., Sickel H., Norderhaug A., Sickel M., and Vangen O. (2006). Plant and vegetation preferences for a high and a moderate yielding Norwegian dairy cattle breed grazing semi-natural mountain pastures. *Anim. Res.* **55**, 367–387. doi:10.1051/animres:2006033

Sant'Anna A. C., da Costa M. J., Pascoa A. G., Magalhaes Silva L. C., and Jung J. (2015). Assessing land use by cattle in heterogeneous environments. *Cienc. Rural* **45**, 470–473. doi:10.1590/0103-8478cr20131576

Šárová R., Špinka M., Panamá J. L. A., and Šimeček P. (2010). Graded leadership by dominant animals in a herd of female beef cattle on pasture. *Anim. Behav.* **79**, 1037–1045. doi:10.1016/j.anbehav.2010.01.019

Sather B. C., Kallenbach R. L., Sexten W. J., and Bradley K. W. (2013). Evaluation of Cattle Grazing Distribution in Response to Weed and Legume Removal in Mixed Tall Fescue (Schedonorus phoenix) and Legume Pastures. *Weed Technol.* **27**, 101–107. doi:10.1614/wt-d-12-00075.1

Sawalhah M. N., Cibils A. F., Hu C., Cao H., and Holechek J. L. (2014). Animal-Driven Rotational Grazing Patterns on Seasonally Grazed New Mexico Rangeland. *Rangel. Ecol. Manag.* **67**, 710–714. doi:10.2111/REM-D-14-00047.1

Sawalhah M. N., Cibils A. F., Maladi A., Cao H., Vanleeuwen D. M., Holechek J. L., Rubio C. M. B., Wesley R. L., Endecott R. L., Mulliniks T. J., and Petersen M. K. (2016). Forage and weather influence day versus nighttime cow behavior and calf weaning weights on rangeland. *Rangel. Ecol. Manag.* **69**, 134–143. doi:10.1016/j.rama.2015.10.007

Schlecht E., Hiernaux P., Kadaouré I., Hülsebusch C., and Mahler F. (2006). A spatio-temporal analysis of forage availability and grazing and excretion behaviour of herded and free grazing cattle, sheep and goats in Western Niger. *Agric. Ecosyst. Environ.* **113**, 226–242. doi:10.1016/j.agee.2005.09.008

Schlecht E., Hulsebusch C., Mahler F., and Becker K. (2004). The use of differentially corrected global positioning system to monitor activities of cattle at pasture. *Appl. Anim. Behav. Sci.* **85**, 185–202. doi:10.1016/j.applanim.2003.11.003

Schoenbaum I., Kigel J., Ungar E. D., Dolev A., and Henkin Z. (2017). Spatial and temporal activity of cattle grazing in Mediterranean oak woodland. *Appl. Anim. Behav. Sci.* **187**, 45–53. doi:10.1016/j.applanim.2016.11.015

Schwarte K. A., Russell J. R., and Morrical D. G. (2011). Effects of pasture management and off-stream water on temporal/spatial distribution of cattle and stream bank characteristics in cool-season grass pastures. *J. Anim. Sci.* **89**, 3236–3247. doi:10.2527/jas.2010-3594

Sickel H., Ihse M., Norderhaug A., and Sickel M. A. K. (2004). How to monitor semi-natural key habitats in relation to grazing preferences of cattle in mountain summer farming areas - An aerial photo and GPS method study. *Landsc. Urban Plan.* **67**, 67–77. doi:10.1016/S0169-2046(03)00029-X

Spedener M., Tofastrud M., Devineau O., and Zimmermann B. (2019). Microhabitat selection of free-ranging beef cattle in south-boreal forest. *Appl. Anim. Behav. Sci.* **213**, 33–39. doi:10.1016/j.applanim.2019.02.006

Spiegal S., Estell R. E., Cibils A. F., James D. K., Peinetti H. R., Browning D. M., Romig K. B., Gonzalez A. L., Lyons A. J., and Bestelmeyer B. T. (2019). Seasonal Divergence of Landscape Use by Heritage and Conventional Cattle on Desert Rangeland. *Rangel. Ecol. Manag.* **72**, 590–601. doi:10.1016/j.rama.2019.02.008

Sprinkle J. E., Taylor J. B., Clark P. E., Hall J. B., Strong N. K., and Roberts-Lew M. C. (2020). Grazing behavior and production characteristics among cows differing in residual feed intake while grazing late season Idaho rangeland. *J. Anim. Sci.* **98**, 1–9. doi:10.1093/jas/skz371

Stephenson M. B., and Bailey D. W. (2017). Do movement patterns of GPS-tracked cattle on extensive rangelands suggest independence among individuals? *Ariculture-Basel* **7**, 58. doi:10.3390/agriculture7070058

Thomas D. T., Wilmot M. G., Kelly R. W., and Revell D. K. (2011). Adaptation behaviour of local and rangeland cattle relocated to a temperate agricultural pasture. *Anim. Prod. Sci.* **51**, 1088–1097. doi:10.1071/AN11044

Thompson D. J., Wheatley B. J., Church J. S., Newman R., and Walker J. (2015). Comparing grazing and resting electivity of beef cattle for BC bunchgrass communities using GPS collars. *Can. J. Anim. Sci.* **95**, 499–507. doi:10.4141/CJAS-2014-116

Tofastrud M., Devineau O., and Zimmermann B. (2019). Habitat selection of free-ranging cattle in productive coniferous forests of south-eastern Norway. *For. Ecol. Manage.* **437**, 1–9. doi:10.1016/j.foreco.2019.01.014

Tofastrud M., Hessle A., Rekdal Y., and Zimmermann B. (2020). Weight gain of free-ranging beef cattle grazing in the boreal forest of south-eastern Norway. *Livest. Sci.* **233,** 103955. doi:10.1016/j.livsci.2020.103955

Tomkins N., and O'Reagain P. (2007). Global positioning systems indicate landscape preferences of cattle in the subtropical savannas. *Rangel. J.* **29**, 217–222. doi:10.1071/RJ07024

Tomkins N. W., O'Reagain P. J., Swain D., Bishop-Hurley G., and Charmley E. (2009). Determining the effect of stocking rate on the spatial distribution of cattle for the subtropical savannas. *Rangel. J.* **31**, 267–276. doi:10.1071/RJ07070

Turner L. W., Udal M. C., Larson B. T., and Shearer S. A. (2000). Monitoring cattle behavior and pasture use with GPS and GIS. *Can. J. Anim. Sci.* **80**, 405–413. doi:10.4141/A99-093

Watanabe N., Umemura K., Sakanoue S., Kozakai T., and Kawamura K. (2010). Utilization by cattle of a pasture including aged hilly sections. *Grassl. Sci.* **56**, 160–167. doi:10.1111/j.1744-697X.2010.00189.x

Wesley R. L., Cibils A. F., Mulliniks J. T., Pollak E. R., Petersen M. K., and Fredrickson E. L. (2012). An assessment of behavioural syndromes in rangeland-raised beef cattle. *Appl. Anim. Behav. Sci.* **139**, 183–194. doi:10.1016/j.applanim.2012.04.005

Wyffels S. A., Boss D. L., Sowell B. F., DelCurto T., Bowman J. G. P., and McNew L. B. (2020). Dormant season grazing on northern mixed grass prairie agroecosystems: Does protein supplement intake, cow age, weight and body condition impact beef cattle resource use and residual vegetation cover? *PLoS One* **15**, 1–25. doi:10.1371/journal.pone.0240629

Wyffels S. A., Petersen M. K., Boss D. L., Sowell B. F., Bowman J. G. P., and McNew L. B. (2019). Dormant Season Grazing: Effect of Supplementation Strategies on Heifer Resource Utilization and Vegetation Use. *Rangel. Ecol. Manag.* **72**, 878–887. doi:10.1016/j.rama.2019.06.006

Zengeya F. M., Murwira A., and de Garine-Witchatitsky M. (2015). Inference of herder presence from GPS collar data of semi-free range cattle. *GEOCARTO Int.* **30**, 905–918. doi:10.1080/10106049.2015.1004129

Zengeya F. M., Mutanga O., and Murwira A. (2013). Linking remotely sensed forage quality estimates from WorldView-2 multispectral data with cattle distribution in a savanna landscape. *Int. J. Appl. EARTH Obs. Geoinf.* **21**, 513–524. doi:10.1016/j.jag.2012.07.008
